# Where’s the Noise? Key Features of Spontaneous Activity and Neural Variability Arise through Learning in a Deterministic Network

**DOI:** 10.1371/journal.pcbi.1004640

**Published:** 2015-12-29

**Authors:** Christoph Hartmann, Andreea Lazar, Bernhard Nessler, Jochen Triesch

**Affiliations:** 1 Frankfurt Institute for Advanced Studies, Johann Wolfgang Goethe University, Frankfurt am Main, Germany; 2 International Max Planck Research School for Neural Circuits, Max Planck Institute for Brain Research, Frankfurt am Main, Germany; 3 Ernst Strüngmann Institute (ESI) for Neuroscience in Cooperation with Max-Planck-Society, Frankfurt am Main, Germany; University of Tübingen and Max Planck Institute for Biologial Cybernetics, GERMANY

## Abstract

Even in the absence of sensory stimulation the brain is spontaneously active. This background “noise” seems to be the dominant cause of the notoriously high trial-to-trial variability of neural recordings. Recent experimental observations have extended our knowledge of trial-to-trial variability and spontaneous activity in several directions: 1. Trial-to-trial variability systematically decreases following the onset of a sensory stimulus or the start of a motor act. 2. Spontaneous activity states in sensory cortex outline the region of evoked sensory responses. 3. Across development, spontaneous activity aligns itself with typical evoked activity patterns. 4. The spontaneous brain activity prior to the presentation of an ambiguous stimulus predicts how the stimulus will be interpreted. At present it is unclear how these observations relate to each other and how they arise in cortical circuits. Here we demonstrate that all of these phenomena can be accounted for by a deterministic self-organizing recurrent neural network model (SORN), which learns a predictive model of its sensory environment. The SORN comprises recurrently coupled populations of excitatory and inhibitory threshold units and learns via a combination of spike-timing dependent plasticity (STDP) and homeostatic plasticity mechanisms. Similar to balanced network architectures, units in the network show irregular activity and variable responses to inputs. Additionally, however, the SORN exhibits sequence learning abilities matching recent findings from visual cortex and the network’s spontaneous activity reproduces the experimental findings mentioned above. Intriguingly, the network’s behaviour is reminiscent of sampling-based probabilistic inference, suggesting that correlates of sampling-based inference can develop from the interaction of STDP and homeostasis in deterministic networks. We conclude that key observations on spontaneous brain activity and the variability of neural responses can be accounted for by a simple deterministic recurrent neural network which learns a predictive model of its sensory environment via a combination of generic neural plasticity mechanisms.

## Author Summary

Neural recordings seem very noisy. If the exact same stimulus is shown to an animal multiple times, the neural response will vary substantially. In fact, the activity of a single neuron shows many features of a random process. Furthermore, the spontaneous activity occurring in the absence of any sensory stimulus, which is usually considered a kind of background noise, often has a magnitude comparable to the activity evoked by stimulus presentation and interacts with sensory inputs in interesting ways. Here we show that the key features of neural variability and spontaneous activity can all be accounted for by a simple and completely deterministic neural network learning a predictive model of its sensory inputs. The network’s deterministic dynamics give rise to structured but variable responses matching key experimental findings obtained in different mammalian species with different recording techniques. Our results suggest that the notorious variability of neural recordings and the complex features of spontaneous brain activity could reflect the dynamics of a largely deterministic but highly adaptive network learning a predictive model of its sensory environment.

## Introduction

Our brains are always active, even when we rest or sleep. This may seem somewhat surprising given the high metabolic costs associated with neural activity [1]. It seems there should be some important function associated with such spontaneous brain activity to justify the associated metabolic expense. It might be that keeping the brain spontaneously active is much like keeping the engine of your car running while waiting at a red light—allowing you to take off faster once the light turns green. On the other hand, without the constantly reverberating neural activity, wouldn’t we be like automata that only jump to action if triggered by an external event? Maybe spontaneous brain activity, rather than being some form of “noise” that keeps the engine running, is the very core on which our minds are constructed.

The problem of spontaneous activity is closely tied to the notoriously high trial-to-trial variability of cortical responses to identical stimuli [2]. Such variability is commonly interpreted as resulting from internal noise. However, while neurons seem to be quite noisy under frequently used laboratory conditions (DC input currents and room temperature), the noise is absent [3] or reduced [4–6] in more realistic conditions, and cannot account for the full trial-to-trial variability of neural responses [7,76]. Instead, spontaneous activity prior to stimulus onset seems to be the underlying cause of trial-to-trial variability. This was shown by Arieli et al. in a seminal study [8] almost 20 years ago using voltage-sensitive dye imaging (VSDI). They demonstrated that trial-to-trial variability can be almost perfectly predicted from the spontaneous activity prior to stimulus onset through a simple linear combination of the spontaneous activity prior to stimulus onset and the stimulus-triggered-average response. They concluded that “the effect of a stimulus might be likened to the additional ripples caused by tossing a stone into a wavy sea.” This encouraged many follow-up studies shedding new light on the relation between spontaneous activity and evoked sensory responses (see [9] for review). Of particular interest are the following four recent findings:

1. Trial-to-trial variability in neural recordings systematically decreases following the onset of a sensory stimulus or the start of a motor act [10]: The authors of this study analysed recordings from seven different macaque brain regions such as V1, MT, or PMd and found a significant decrease in variability with stimulus onset for all of the ten different data sets they considered. Variability was measured as the Fano factor (variance/mean) over trials for each neuron and condition. A number of additional studies suggest that attention has a similar effect in both primates and rodents (see [11] for review).
2. These highly variable spontaneous activity states in sensory cortex outline the region of evoked sensory responses. One of the first studies demonstrating this analysed VSDI data from cat visual cortex during stimulation with gratings [12]. They found that individual frames of the spontaneous and evoked activity had almost the same amplitude and a similarly good match to the averaged orientation maps. In a later study, rats were presented with natural tones and pure tones while recording auditory cortex activity with tetrodes [13]. The authors found that spontaneous activity outlined the region of evoked sensory responses produced by natural sounds and pure tones.
3. This alignment between spontaneous activity and typical evoked activity patterns is increasing during development. There is accumulating evidence that sensory stimulation has a lasting effect on spontaneous activity [14, 15], most probably through learning. This effect can also be tracked over development: [16] compared spontaneous multi-unit activity of ferret V1 to evoked activity in response to natural and artificial stimuli at different ages. They found that the match between spontaneous and evoked activity increases for all stimuli over development but increases most for natural stimuli. A recent control with blindfolded ferrets corroborates this finding [17]. Interestingly, [12] had already shown that activity patterns in cat visual cortex corresponding to horizontal and vertical orientations occur more frequently in spontaneous activity—matching their prominence in natural scenes [18].
4. The spontaneous brain activity prior to the presentation of an ambiguous stimulus predicts how the stimulus will be interpreted. This was demonstrated for both monkeys [19] and humans [20]. In the latter study, subjects were shown an ambiguous face-vase stimulus while their brain activity was monitored with fMRI. Interestingly, the subject’s decisions (face or vase) could be predicted from activity in the fusiform face area (FFA) prior to stimulus onset. These findings complement the one by [8] showing that evoked VSDI responses in cat visual cortex can be predicted almost perfectly from the spontaneous activity prior to stimulus onset and the average response to the stimulus. Taken together, these studies suggest that spontaneous activity can bias perceptual decisions.

The findings on neural variability and spontaneous activity discussed above raise three important questions. First, do these findings reflect independent phenomena or are they somehow related? Second, what neurophysiological mechanisms give rise to these findings? Third, is there a functional meaning to these findings and, if so, what is it?

There are two complementary ways of approaching these questions: bottom-up and top-down. A bottom-approach may try to identify a minimal network model whose mechanisms are modelled after biological findings that is able to reproduce the observed effects and explain how they are rooted in basic physiological mechanisms. A top-down or normative approach will start with an assumed function and investigate whether and how (approximately) optimal solutions to this function resemble the observed physiological findings. In the end, the goal is always to reconcile both views into a unified picture. Our approach follows the bottom-up route.

We hypothesize that three properties of cortical circuits might underlie all these findings on spontaneous activity and neural variability:

1. Recurrent connectivity shapes the structure of spontaneous activity and determines the relationship between spontaneous and evoked activity patterns.
2. Neural plasticity is responsible for structuring recurrent connectivity such that spontaneous activity matches the statistics of evoked activity. In functional terms this corresponds to the network learning a predictive model of its sensory environment.
3. Homeostatic mechanisms keep spontaneous and evoked activity in a healthy dynamic regime where learning and inference are possible.

Evidence for the first property comes from a range of studies. First of all, neural structures that do not receive recurrent input, such as the retina, display much lower response variability [21, 22] suggesting that neural variability is induced by fluctuations of recurrent input. Second, there is anatomical evidence that V1 receives less than 5% bottom-up input synapses and more than 95% of synapses stem from neurons within V1 and higher-level areas [23]. Retrograde labelling and paired recordings show that neurons responding to the same grating or natural stimulus are both more likely to be connected and have stronger connections than expected by chance, suggesting that these recurrent connections enforce specific neural populations to be coactive [24, 25]. Another link between anatomy and function was obtained by comparing long-distance axonal projections and spontaneous activity patterns [26]. The authors found that spontaneous and evoked activity followed the axonal pathways. Similar results were obtained by comparing resting state activity in fMRI and anatomical pathways determined by diffusion tensor imaging [27]. Finally, purely functional studies show that the order of activation of individual cells is similar during spontaneous and evoked activity suggesting that it is constrained by the underlying recurrent connectivity [28]. Taken together, there is broad evidence that recurrent connectivity shapes the common structure of spontaneous and evoked activity.

Second, evidence from developmental studies suggests that the statistics of features in the visual world get translated into cortical connectivity by means of Hebbian plasticity [29, 30]. A recent study demonstrates that such self-organization is still present in adult animals as early as in V1 [31]: In this experiment, adult mice were stimulated with repeated sequences of gratings of different orientations. The authors then recorded activity from neurons in V1 and found that the circuit formed a predictive representation of these spatiotemporal sequences. In particular, the circuit “recognized” a sequence by responding stronger to it than to the reversed sequence and showed predictive behaviour by “filling in” activity when parts of the sequence were left out. Pharmacological controls suggest that the underlying plasticity was local to V1.

Together with the observation that the statistics of spontaneous activity match the statistics of the environment of the organism [12, 14–16], this suggests that the statistics of the environment are stored in the recurrent connectivity of the neural circuit and that the structure of spontaneous activity is simply an image of the learnt circuit structure that reveals itself when the network receives no external drive.

Evidence for the third property comes mainly from computational neuroscience studies. While it is known that homeostatic plasticity is essential to restore a balance between excitation and inhibition after perturbation by different forms of input deprivation (reviewed in [32]), there are only few experimental studies on the direct interaction between homeostasis and learning (see, e.g., [33, 34]). Theoretical studies on neural plasticity, on the other hand, have long argued that homeostasis is needed to “tame the beast” of Hebbian learning by enforcing stable network dynamics [35]. These theoretical predictions are consistent with clinical findings from different brain pathologies. For example, dysfunctional homeostasis has been linked to autism spectrum disorder and intellectual disabilities [36].

To test if these three properties are indeed sufficient to account for the many findings on spontaneous activity and neural variability, we capture them in a minimal neural network model: The self-organizing recurrent neural network (SORN, [37]) is a network of interconnected excitatory and inhibitory populations of McCulloch & Pitts model neurons. These neurons have binary states determined by thresholding the input, are updated synchronously, and are fully deterministic. The excitatory neurons are recurrently connected and receive structured input sequences. A binarized form of spike-timing dependent plasticity (STDP) shapes these recurrent excitatory connections. The network dynamics are kept in check by two forms of homeostatic plasticity: Synaptic normalization ensures that the summed synaptic weights from and onto each neuron stay the same during STDP. Intrinsic plasticity regulates the thresholds of the excitatory neurons on a slow timescale to avoid silent or overly active neurons. SORN networks have been shown to have powerful sequence learning abilities. For example, on challenging sequence learning tasks involving hidden states and long temporal dependencies they greatly outperform analogous reservoir architectures without plasticity in the recurrent network [37]. More recently, they have also been shown to produce state-of-the-art results in learning artificial grammars with their performance resembling that of human subjects [38]. Furthermore, such networks have also been shown to capture experimental data on the statistics and fluctuations of synaptic efficacies [39]. These features make them interesting candidates for modelling experimental findings on spontaneous activity and neural variability.

In the following we show how the SORN model reproduces the key findings on spontaneous activity and neural response variability discussed above. To capture the essence of the experimental situations above, we define two learning tasks: a *sequence learning task* and an *inference task*. Both are deliberately simple compared to previous SORN studies [37, 38] but suffice to capture the key findings. The *sequence learning task* is inspired by [31] and consists of presenting the network with one or more discrete sequences of input stimuli. After replicating this study, which demonstrated the learning of predictive models in V1 of adult mice, we use similar tasks to study the similarity of spontaneous and evoked activity. The *inference task* is inspired by [20], where subjects are presented with an ambiguous face-vase stimulus and their FFA activity is used to predict their perceptions. Apart from replicating this specific result, the trial structure of this task allows us to study the effect of spontaneous activity on evoked activity.

By carefully analysing the SORN’s behaviour in these two tasks, we show how it readily reproduces the key experimental findings on spontaneous activity and neural variability without requiring any internal noise. On the other hand, adding small amounts of noise to the network produces similar findings (see Discussion). Taken together, our results suggest that the experimental findings on neural variability reflect the dynamics of a largely deterministic but highly adaptive cortical network learning a predictive model of its sensory environment. Our findings also support the claim that the notorious variability of neural responses is largely due to spontaneous network dynamics. It is noteworthy that a simple model such as the SORN can account for such a diverse set of findings that were independently obtained in a range of different species with a range of different recording techniques (multi-electrode and single cell recordings, optical imaging, functional magnetic resonance imaging) in a number of different labs. It suggests that the SORN model as a minimal instantiation of the three properties introduced above captures some fundamental principles of cortical learning and information processing in a distilled form.

## Results

### Basic model properties

We begin by characterizing some dynamic properties of the network. For this, we stimulate the network with random alternations of ten letters, where each letter presentation corresponds to stimulating a subset of excitatory neurons (see Methods for a detailed description of the model and stimulation paradigms). After the fraction of excitatory to excitatory connections converges, we deactivate STDP and stop stimulation to observe the spontaneous activity. This spontaneous activity develops as the intrinsic plasticity of each neuron shifts the excitability thresholds to compensate for the missing input.

**Fig 1:**
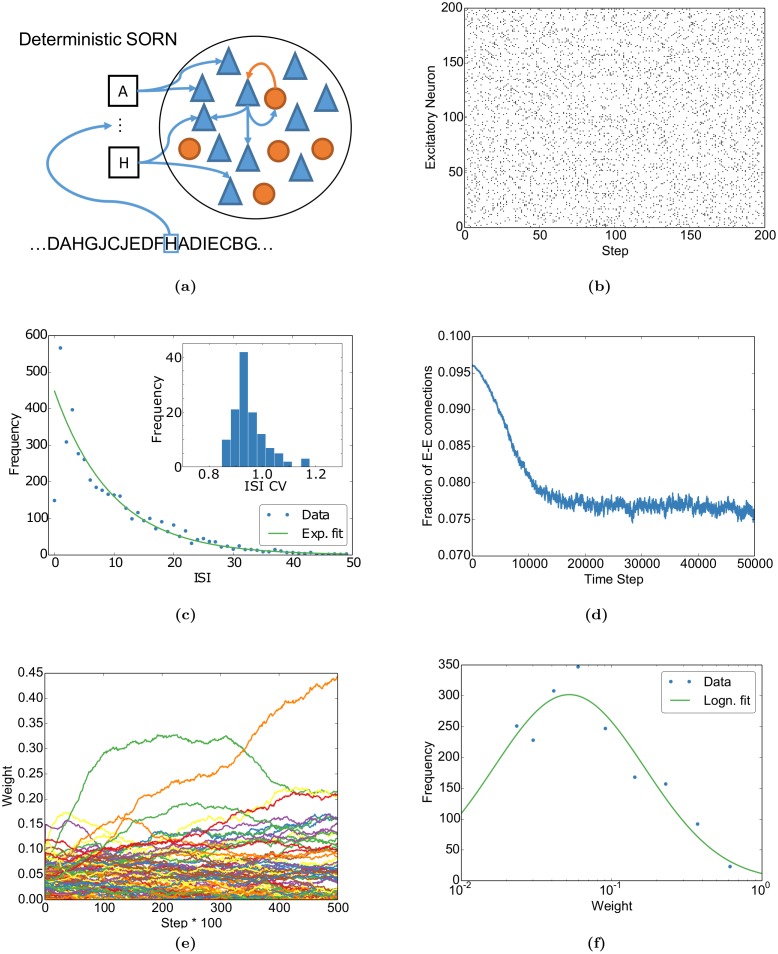
Basic properties of the network match experimental findings. (a) The Self-Organizing Recurrent Neural Network (SORN) consists of recurrently connected excitatory (blue) and inhibitory (red) deterministic McCulloch & Pitts threshold neurons. Each input letter (black boxes) stimulates an excitatory subpopulation. The excitatory recurrent connections are shaped by spike-timing dependent plasticity and synaptic normalization. The excitatory thresholds are regulated by intrinsic plasticity (see Methods for details). (b) Raster plot of spontaneous activity (no external input) after stimulating the network with ten randomly alternating letters during plasticity. (c) The inter-spike-interval (ISI) distribution of a randomly selected neuron during spontaneous activity is well-fitted by an exponential apart from very small ISIs. (c, inset) The distribution of coefficients of variation (CVs) of the ISIs clusters around one, as expected for exponential ISI distributions, compatible with the experimentally observed Poisson-like spiking [10, 40]. (d) The fraction of excitatory-to-excitatory connections converges to a stable fraction. (e) Individual weights fluctuate despite the global convergence as observed experimentally [41]. (f) After self-organization, i.e. at the end of (d), the binned distribution of excitatory-to-excitatory synaptic weights (dots) is well fit by a lognormal distribution (solid line, cp., e.g., [42]).

Fig 1b and 1c shows that the activity of individual neurons of the network is well described by an exponential inter-spike interval (ISI) distribution and can be summarized by a coefficient of variation distributed around 1. These indicate that the individual neurons fire irregularly with Poissonian statistics, a key feature of neural variability in neocortex [10, 40]. Additionally, while the network converges to a stable fraction of connections, the individual weights keep fluctuating (Fig 1d and 1e) as observed experimentally [41]. Finally, the network approaches a lognormal-like weight distribution after learning (Fig 1f), which is a documented property of cortical circuits [42]. Taken together, these results demonstrate that some essential features of neocortex taken as evidence for a “noisy” brain are readily captured by our deterministic model.

### Learning spatiotemporal sequences

Next, we replicate the sequence learning paradigm from [31]. In their study, the authors stimulated mice with a discrete set of gratings of different orientations. For example, they stimulated their mice with the sequence “ABCD”, which refers to four distinct gratings presented in succession. They then recorded activity from neurons in V1 and found that the circuit formed a representation of these spatiotemporal sequences.

We modelled this experiment by stimulating our network with a similar set of stimuli. We model visual input to V1 by stimulating a small subpopulation of our excitatory units when a stimulus is presented. This subpopulation can be thought of as having receptive fields that align with the grating in the original study.

**Fig 2:**
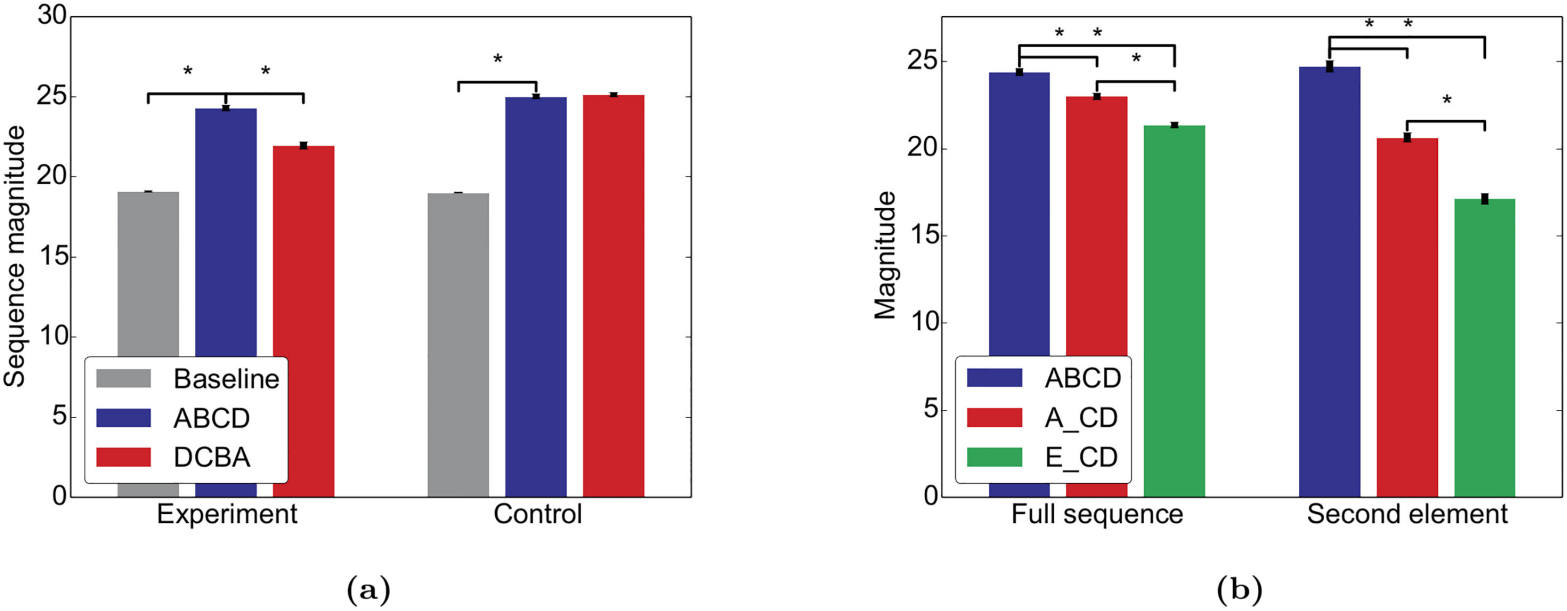
The network learns a predictive model mimicking [31]. Similar to [31], we stimulated the network either with the sequence “ABCD” (experiment) or with all permutations of this sequence (control). After a period of self-organization, the network was tested with the sequences “ABCD” and “DCBA”. (a) Mean firing rates (“sequence magnitude”) for both test sequences. (b) For the experimental condition, the network was also tested with the sequences “ABCD”, “A_CD”, and “E_CD”. Error bars represent SEM over 20 independent realizations. ⋆ indicates *p* < 0.05 for a two-sided t-test assuming independent samples and identical variances.

To replicate the first experiment from [31], we stimulated our network with the sequence “ABCD” during an initial period of self-organization. After the stimulation, we deactivated STDP, stopped the stimulation, and ran the network only with intrinsic plasticity to get a period of spontaneous activity, similar to the sleep phase in the original study. Finally, we tested the network with either “ABCD” (same sequence) or “DCBA” (reversed sequence). The network matched the effects reported in Fig 1 of [31] in that it responded with a higher mean rate to “ABCD” than to “DCBA” indicating that the trained sequence was “recognized” (Fig 2a). In a control experiment we trained the network on all permutations of the original sequence (i.e. “ABCD”, “ABDC”, …, “DCBA”) and similar to the matching control from [31], the mean rate was similar for both test sequences.

In a further experiment in [31], the authors then showed that V1 can “predict” upcoming sequence items by training the mice with “ABCD” and testing with “ABCD”, “A_CD” and “E_CD”. They showed that V1 responded more strongly to the first two test sequences than to the third one and more strongly to a “B” or a blank following an “A” than to the blank after an “E” (cp. Fig 4 of [31]). As before, our simulations qualitatively replicate these results (Fig 2b). Note that much more sophisticated sequence learning abilities in SORN have already been demonstrated in [37, 38].

These two experiments demonstrate that our simple model captures key experimental findings on the physiology of sequence learning. With this as a backdrop, we can investigate the properties and interactions of spontaneous and evoked activity in similar learning experiments in the next sections.

### Structured spontaneous activity

Spontaneous activity is structured in space and time [12, 13] and revisits those states, i.e. neuronal activation patterns, more often that correspond to overrepresented features in natural stimuli, such as horizontal and vertical bars in [12]. It has been proposed that this is a result from adapting the spontaneous activity to the statistics of the evoked activity during development [16].

In order to model these findings, we adapted the sequence learning paradigm: During an initial self-organization phase, we stimulate the network with random alternations of two sequences: “ABCD” and “EFGH” with different relative probabilities. In a second phase, we deactivate STDP to study the evoked activity of the adapted network. Finally, we stop the input and observe the network’s spontaneous activity. Due to the intrinsic plasticity in the model, our network compensates for the missing input and produces spontaneous activity in the absence of noise.

#### Spontaneous activity is structured in space and time

**Fig 3:**
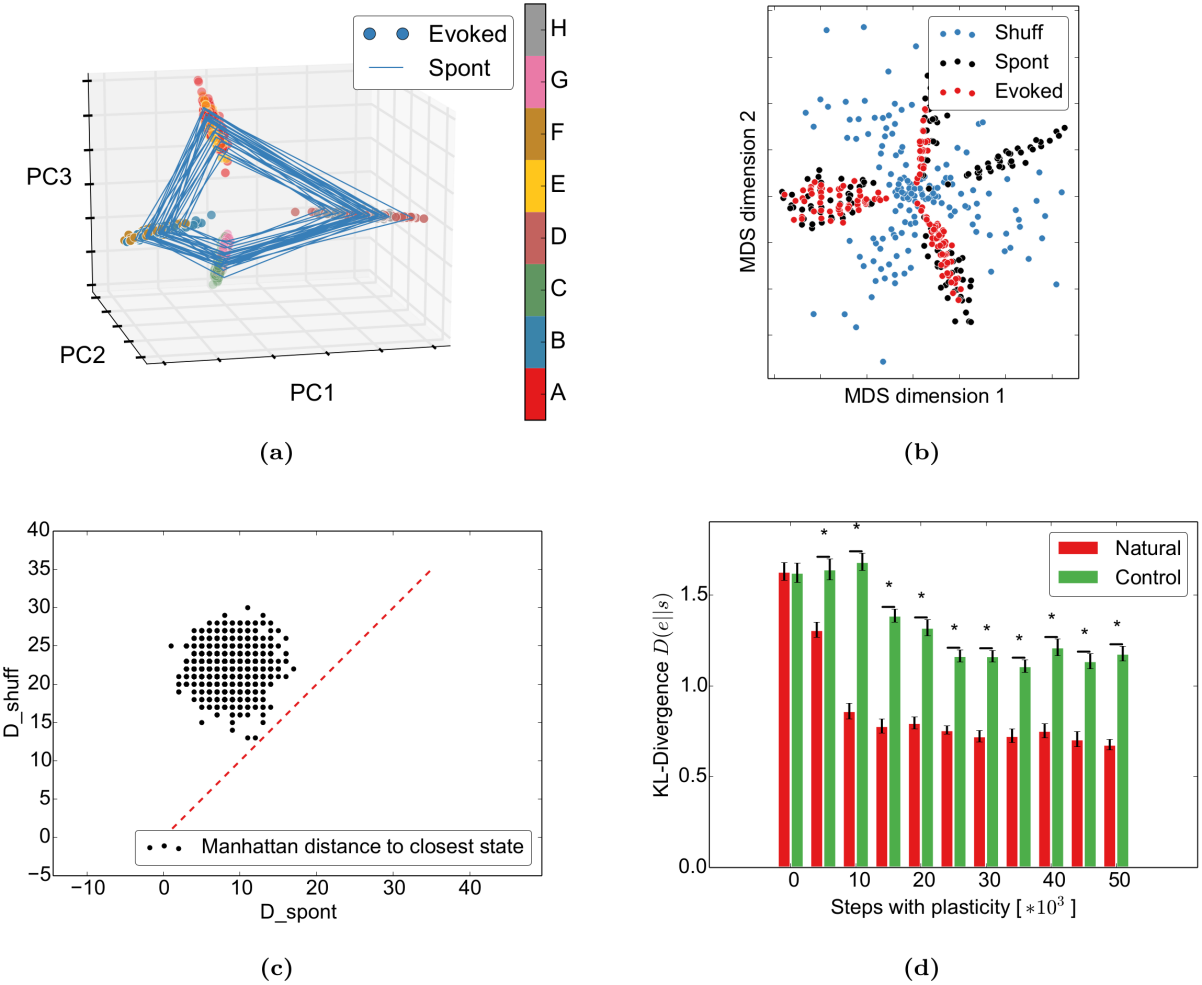
Spontaneous and evoked activity align through self-organization. The network was stimulated with the words “ABCD” (67%) and “EFGH” (33%). (a) The spontaneous activity follows the spatiotemporal trajectories of the evoked states in the PCA projection. (b) In the multidimensional scaling projection, the evoked activity (red) follows the spontaneous outline (black) and avoids the shuffled spontaneous states (blue) (cp. Fig 6c of [13]). (c) The evoked states are closer to the spontaneous states than to the shuffled spontaneous states: As in Fig 6 of [13], the distance from evoked states to the closest spontaneous states (D_spont) is smaller than the distance to the closest shuffled spontaneous state (D_shuff). The red dashed line shows equality. (d) Spontaneous activity becomes more similar to evoked activity during learning: After self-organizing to “ABCD” and “EFGH” with identical probabilities, spontaneous activity was compared to the evoked activity from the imprinted sequences (natural) or the two control sequences “EDCBA” and “FGH” (control) with Kullback-Leibler divergence. New networks were generated for each training time and condition. Error bars represent SEM over 50 independent realizations. ⋆ indicates *p* < 0.05 for a t-test assuming independent samples and identical variances.

As a first step, we want to get a better understanding of the spontaneous network dynamics by visualizing the activity of the 200 simulated excitatory neurons in three dimensions. For this, we apply principal component analysis to project the 200-dimensional spontaneous and evoked binary activity vectors to the first three principal components (PCs) of the evoked activity. These three components usually account for 40–50% of the variability. The evoked activity (points) captures the properties of the input by forming one activity cluster for each position in the input sequences (“A” and “E”, “B” and “F”, …) (Fig 3a). We believe that the very similar transition structure is responsible for this: both “D” and “H” transition to either “A” or “E”. Since the neurons coding for “D” and “H” will only have a small subset of overlapping projections but these postsynaptic neurons have to code for both “A” or “E”, “A” and “E” should look similar. A similar argument applies for the following letters. Despite the overlap in the first PCs, both sequences can be clearly separated in later PCs (S3 Fig). We observe a trend that letters later in the word separate “easier”, i.e. in earlier PCs. For example, “H” and “D” separate in PC4, “G” and “C” in PC9, “B” and “F” in PC11, and “A” and “E” in PC 15 for this simulation. This is probably due to small differences at the beginning accumulating through the recurrent structure to larger differences towards the end of the word. In addition to the learnt structure, the spontaneous activity (lines) closely follows the structure of the evoked activity. This observed spontaneous replay of evoked sequences is similar to [12, 14, 43]. The authors of [12] showed with optical imaging in cat area 18 that spontaneous activity is highly structured and smoothly varies over time (in their case it smoothly switches between neighbouring orientations). They also observe that spontaneous activity preferentially visits states that correspond to features that occur more often in nature (in their case horizontal and vertical bars). We demonstrate an abstract version of the latter point below.

#### Spontaneous activity outlines sensory responses

Next, we tested if the SORN model captures the finding of [13] that “spontaneous events outline the realm of possible sensory responses”. To model the conditions of the original experiment, we compared spontaneous activity to evoked activity from only 5 randomly selected letters from the original words. This captures the fact that only a subset of the “lifetime experience” of stimuli was presented during the experiment. As in [13], 150 randomly selected spontaneous activity vectors of the excitatory units, their shuffled versions, and 150 of the just described evoked events were then reduced from the high-dimensional activity patterns of excitatory neurons to 2D by multidimensional scaling (MDS). Simply put, this method uses all variability in the data to represent the distances between data points in their high-dimensional space (neural activity vectors) in the plotted two dimensions as well as possible. This is in contrast to the previous 3D-PCA plots, which only consider the variability in the first three principal components. Our results indicate that the spontaneous activity outlines the evoked activity while the shuffled activity does not capture its structure (Fig 3b). Similar to the experimental study, we confirm this by showing that evoked activity states are significantly closer to the spontaneous activity states than to the shuffled spontaneous ones (Fig 3c).

#### Spontaneous activity adapts to evoked activity

Given the previous results, the question arises how these phenomena are related to the network self-organization. For this, we compared the effect of learning to results from [16]. They showed that during development, the distance between the distribution of spontaneous activity and the distribution of evoked activity decreases. Interestingly, the distance decreases both for stimuli that the animal was exposed to during development (natural movies) and to artificial stimuli (gratings and bars), but the reduction is less pronounced for the artificial stimuli. We try to capture the essentials of this experiment by presenting the same two sequences during the self-organization phase. After learning, we then either present the same sequences (natural condition) or the two control sequences “EDCBA” and “HGF” (control condition) for as many steps as the number of bins in the original paper. We chose this specific control condition because it has a different structure while still stimulating the same input units, similar to the control conditions in the original study. In both conditions, the sequences are presented with equal probability. The evoked network states were then compared to the spontaneous states using the KL-divergence. Our model shows a qualitatively similar behaviour in that the KL-divergence between the distribution of evoked responses in the natural condition and the distribution of spontaneous responses decreases during learning (Fig 3d). This decrease is larger compared to the decrease observed for the reversed sequence.

Taken together, these results show that in our simple model the spontaneous network activity outlines the possible sensory responses after self-organization. It is important to note that none of these effects occur in a random network without plasticity (see [37] for sample network dynamics of the SORN without plasticity).

#### Self-organization captures stimulus probabilities

**Fig 4:**
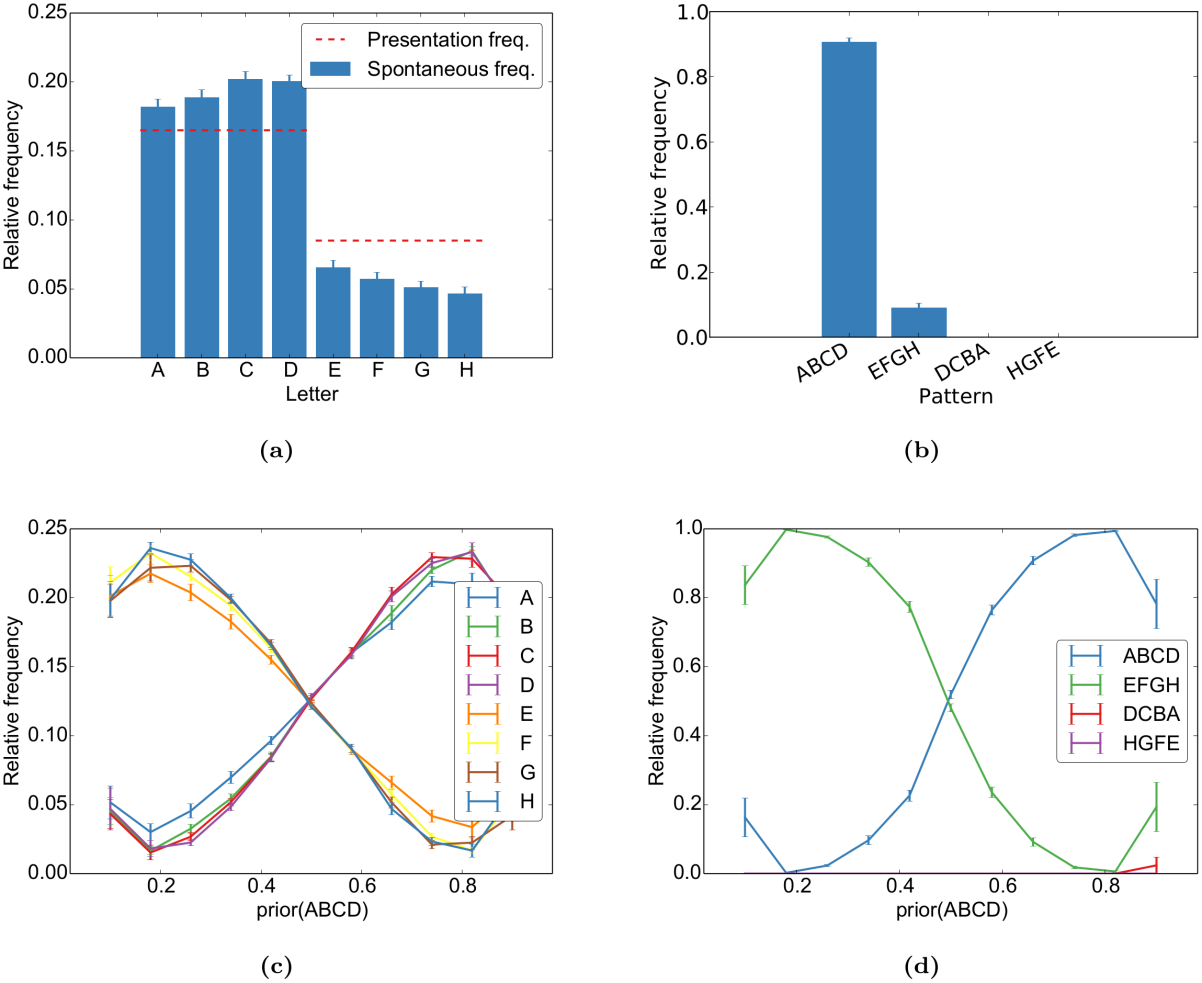
Different priors are learnt by the network. During self-organization, the sequences “ABCD” and “EFGH” were randomly interleaved with frequencies of 67% and 33%, respectively. This is reflected in the relative occurrence of (a) each letter and (b) each word in the spontaneous activity. For different priors during self-organization, this results in the frequencies in (c) for each letter and in (d) for each word. Both show overlearning effects in that their frequencies are biased in favour of the word that was shown more often. The reversing trend and high variance for the extreme priors (0.1 and 0.9) can be accounted for by pathological network dynamics for some simulations with these priors. The letter frequency is the observed frequency in the evoked activity while the word frequency was normalized over the total number of observed words (“ABCD”, “EFGH”, “DCBA”, and “HGFE”) to yield better comparison over different realizations. Error bars represent SEM over 20 independent realizations.

Having compared our model dynamics to qualitative features of spontaneous activity, we next performed a quantitative analysis on how the learnt sequences are represented in the network. In order to do so, we assign to each spontaneous state a stimulus letter based on the stimulus of the closest matching evoked network state (see Methods for details). From this, we compute the frequency of word-occurrence in the spontaneous activity. The spontaneous states resemble the evoked states in two ways (Fig 4a and 4b): First, the letters that were presented more often in the evoked activity also occur more often in the spontaneous activity. Second, the transition between states occurs in the correct temporal direction while reversed transitions rarely occur in the spontaneous activity. By varying the probability of each sequence during self-organization, we can quantify how these priors are captured by the spontaneous activity. As one can see in Fig 4c and 4d, the probability of the words and letters approximate their frequency during learning. However, we observe a tendency to overrepresent the more frequent stimuli. Additionally, the frequency of letters increases towards the end of the more frequent word while it decreases towards the end of the less frequent word. This is due to spontaneous switching between the two words: the more frequent word is more robust and activity switches less often to the less frequent word than the other way round. We further explore this issue in the section Network Analysis below.

### Interaction between spontaneous activity and evoked activity

After observing the properties of spontaneous activity, we next investigate the interaction between spontaneous activity and evoked activity in an inference task with trial-like interleaved spontaneous and evoked activity. By inference task, we mean that the task involves integrating previously acquired knowledge with an ambiguous sensory input to arrive at a unique interpretation of that stimulus. This non-formal usage of the term inference has a long tradition going back at least to Hermann von Helmholtz who used the German expression “unbewusster Schluss”, which is usually translated as unconscious inference.

This task is used to model the studies demonstrating that the neural variability significantly drops at the onset of a stimulus [10], that the evoked activity can be linearly predicted from the spontaneous activity [8], and that the spontaneous activity before stimulus onset predicts the decisions when a noisy [19] or ambiguous [20] stimulus is presented. We model these findings by training our networks on a task inspired by [20]. In their setting, an ambiguous face-vase stimulus is immediately followed by a mask. After the mask, the subjects have to decide whether they perceived a face or a vase. This trial structure allows us to study the interaction between spontaneous activity before stimulus onset and the evoked activity following the presentation of a stimulus.

We model the task as follows: during training, the network is presented with two randomly alternating input sequences represented as “AXXX_ _ _ …” and “BXXX_ _ _ …” with different probabilities. “A” and “B” stand for the face and vase, respectively, the common “XXX” for the mask, and “_ _ _ …” for periods without input corresponding to a blank screen. During the initial self-organization the network learns to represent these sequences and their prior probabilities. After self-organizing to these stimuli, STDP is switched off and a linear classifier is trained to postdict whether “A” or “B” has been presented based on the neural activity at the first blank stimulus, “_”. We define the network “decision” as the postdiction of this classifier for either “A” or “B” similar to subjects’ “decision” whether they just saw a face or a vase. The mask ensures that the decision is based on an internal representation and not simply on a direct input-output mapping. During subsequent testing, the network is stimulated with ambiguous mixtures of “A” and “B”, which we indicate as “A/B”, followed by the mask. Similar to human subjects reporting on their ambiguous percept, the network then has to decide with the classifier for “A” or “B” in light of the ambiguity of “A/B” and the prior probabilities of “A” and “B” during training. Different levels of ambiguity are modelled by stimulating *f*_A_ × 10 input units of “A” and (1 − *f*_A_) × 10 input units of “B” where *f*_A_ is a fraction. Further model details are given in the Methods.

As in the previous section, we will first investigate the qualitative behaviour of the model self-organizing to these stimuli and compare them to the experimental findings. Thereafter, we will describe how the network performs inference for the ambiguous stimuli.

#### Stimulus onset quenches variability

**Fig 5:**
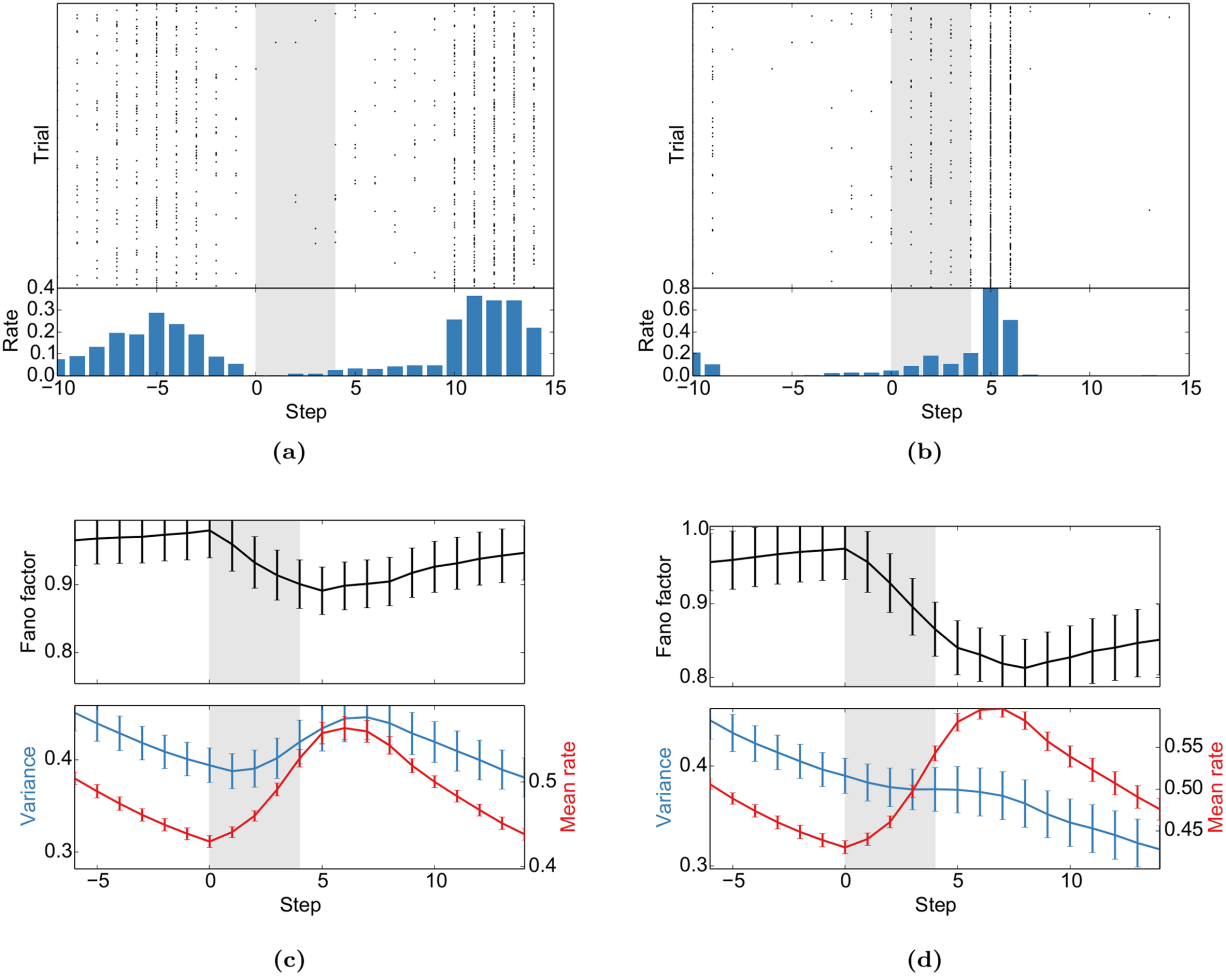
Stimulus onset quenches variability. (a) and (b) Sample spike trains from two representative neurons aligned to the stimulus presentation (shaded area). (c) and (d) The population average of the Fano factor (FF) decreases with stimulus onset. The FF is only computed for units that do not receive direct sensory input. These results mimic Fig 5 of [10]. FFs, mean rates and variances are for causal moving windows of 5 time steps. (c) was computed for the presentation of stimulus “AXXX_ _ _ …” during the test phase after being presented with a probability of 0.1 during self-organization. (d) In turn, “BXXX_ _ _ …” had a probability of 0.9 in the same experiment. Error bars represent SEM over 20 independent realizations.

First, we replicate the “widespread cortical phenomenon” that the “stimulus onset quenches variability” [10]: While the neural activity and the activity in this model is in general highly variable and shows signatures of a Poisson process (cp. Fig 1), we observe a drop in variability measured by the Fano factor (FF) in response to stimulus onset (Fig 5). We also found that this effect is significantly stronger when the respective stimulus had a higher presentation probability (Fig 5c and 5d). Furthermore, the stimulus that was presented more often also elicits a higher mean firing rate. A similar effect was observed for MT responses to moving gratings [44]: the Fano Factor decreased most for the preferred direction, i.e. for the direction with the highest mean rate.

Across all stimuli, the drop of the FF is accompanied by a rise of the mean firing rate (Fig 5c). To control for this, we performed a “mean matching” analysis as suggested in [10] (see Methods for details). S4 Fig shows a mean-matched decrease of the FF at stimulus onset.

#### Spontaneous activity predicts evoked activity and decisions

Next, we test our model on two similar findings: First, the authors of [8] found that the optical imaging response evoked by simple bar stimuli is almost identical to the sum of the spontaneous activity prior to stimulus onset and the average stimulus-triggered response. Second, the original study that we model here [20] found that spontaneous activity prior to stimulus onset predicts the decision of the subjects. Thus, spontaneous activity influences the evoked response at the level of neural responses and behavioural decisions.

We model these experiments by training simple linear classifiers either to predict the evoked spiking of individual cells from the spontaneous activity immediately before stimulus presentation, or to predict the decision of the network after the presentation of stimuli with different ambiguities. These are compared to a baseline prediction from a “control classifier” that is based on shuffled spontaneous activity.

**Fig 6:**
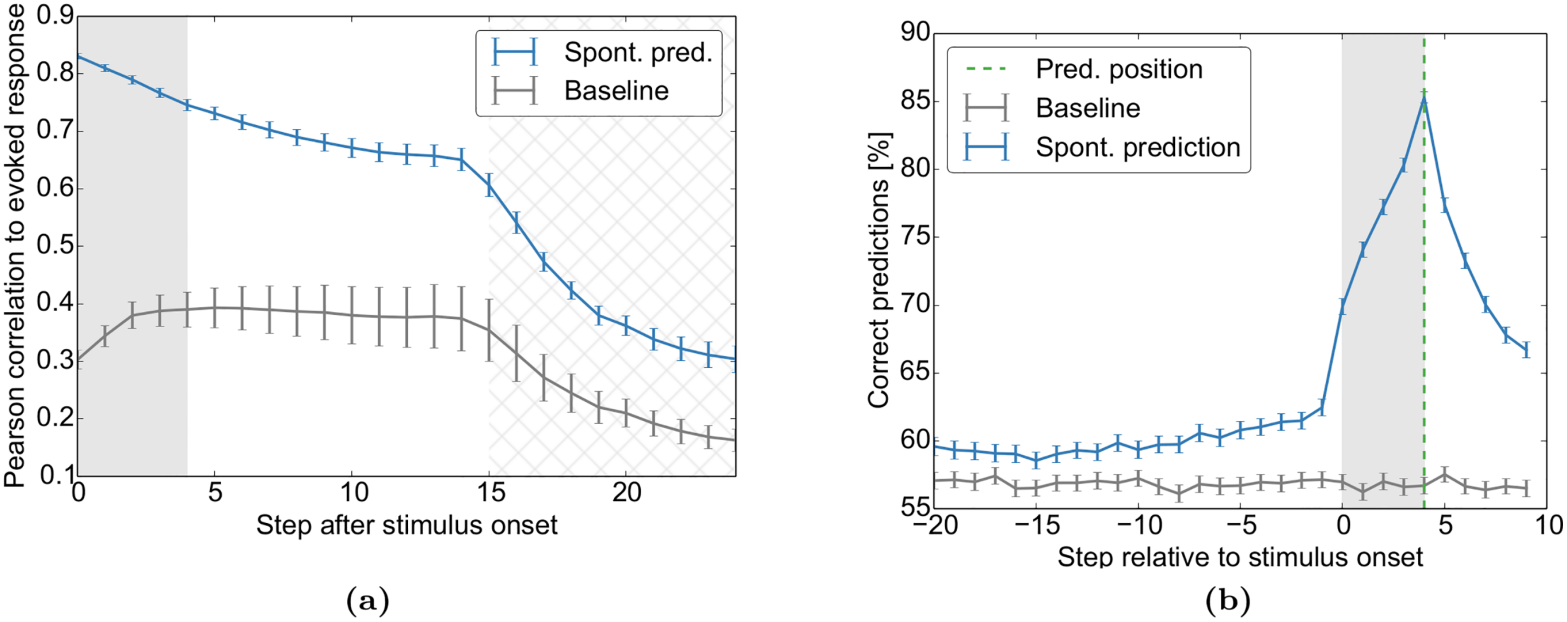
Spontaneous activity predicts evoked activity and decisions. The network self-organizes during repeated presentations of the sequences “AXXX_ _ _ …” (33%) and “BXXX_ _ _ …” (67%) with blank intervals in between. In the test phase, an ambiguous mix of cue “A” and cue “B” is presented (“A/BXXX_ _ _ …”, shaded area) and the network decides at the first “_” with a linear classifier if either A or B was the start of the sequence. (a) Trial-to-trial variability of the evoked activity patterns during the test phase is well predicted from activity prior to stimulus onset. The figure shows the correlation between the variable evoked activity patterns at different time steps after stimulus onset and a linear prediction of these based on the stimulus and either the spontaneous activity state prior to stimulus onset (blue) or trial-shuffled spontaneous activity (baseline, grey). Similar to Fig 4 in [8], the decay of the correlation is exponential-like. The correlation drops further as new stimuli are presented (hatched area). Due to variable inter-trial-intervals, the hatched area covers the entire area were stimulation is possible. (b) The decisions of the network can be predicted from spontaneous activity before stimulus onset. The plot shows the accuracy of predicted network decisions (“A” vs. “B”) at the green dashed line from activity surrounding the decision. Separate classifiers were trained for each of the 11 ambiguity classes (e.g. 20%A) and time step surrounding the decision. The grey line corresponds to predictions from trial-shuffled activity. Predictions in (b) are averaged over all priors of Fig 7c. Error bars represent SEM over 20 independent realizations.

We find that the spontaneous activity prior to stimulus onset allows prediction of the evoked activity (Fig 6a) and the final decision (Fig 6b) in a linear manner. These two complementary experiments demonstrate that the network’s spontaneous activity prior to the stimulus contains significant information about subsequent evoked responses and perceptual decisions as demonstrated experimentally in [8] and [20].

The initial trial-shuffled baseline prediction in Fig 6a follows a trend similar to the inverted Fano Factor (Fig 5): it initially increases and then decreases. This is because a more stereotypical response will be both predicted more easily and have a lower variability. The tail of the prediction then decays further due to the fading of memory in these networks [37]. As new stimuli are presented (hatched area), the predictability drops even further since the next stimulation is independent of the previous one. In experimental settings, where one does not have access to the full network state, the unobserved states would have a similar effect to our new inputs: the predictability would decay due to variability that one does not have access to. So even for purely spontaneous activity after stimulation, one would expect a quick decay of predictability due to inputs to the circuit from brain areas that were not recorded.

Please note that the baseline in Fig 6b is above 50%. This is because the decisions of the network are usually biased towards “A” or “B” similar to human subjects being biased to perceive more faces or vases in the face-vase experiment. This bias can be exploited by the control classifier by always predicting the more likely decision and thereby getting above-50% accuracy. Also note that the stimulus onset triggers an increase in predictability. This is due to the nature of stimulation: for the same ambiguous stimulus, different neurons are stimulated at each trial, which influences the decision to be predicted.

Taken together, all these results show that the complex dynamics of a very simple self-organizing recurrent neural network suffice to reproduce key features of the interaction of spontaneous and evoked activity.

#### Network dynamics is reminiscent of sampling-based inference

The core of Bayesian learning and inference is the probabilistically correct combination of the hitherto observed experience—which we have already identified as prior in the previous spontaneous activity experiment—with a current stimulus *s*, which is the new instantaneous evidence in Bayesian terminology. To which extent does the self-organized learning of our model and the subsequent response evoked by a stimulus reflect the Bayesian inference capabilities of the brain as investigated in a number of experiments (see, e.g. [45], but also see [46])? Specifically, we want to investigate to which extent the input-output behaviour of SORN is reminiscent of sampling-based inference.

Before comparing the behaviour of the SORN to a probabilistic inference process we have to reason about the idea where we locate the stochastic component in the deterministic recurrent network. The definition of the SORN implies that its state is deterministic given the current input *and the full internal state of the previous time step*. From the perspective of an external experimenter, however, the internal state is unknown, which leads to a pseudo-stochastic relation between the actual stimulus and the later readout. In the Methods we derive a probabilistic model that reflects this source of uncertainty by formalizing the input stimulation as noisy binary channels. The probabilistic inference is then modelled as a Naive Bayes classifier that gets its evidences from these noisy channels. Note that the probabilistic model is conceived such that it has no *a priori* knowledge about the labels of the channels or their relation to the population “A” or “B”, but it gains this knowledge from a learning phase similar to the SORN model with its subsequent linear classifier. As the result of the probabilistic model we use the posterior probability of the response variable “A” given the ambiguous stimulus *s* with respect to the learned prior *p*(A), or more precisely the probability function *p*(A|*f*_A_) that directly relates the fraction *f*_A_ of ambiguity in *s* to that posterior probability.

Importantly, we are not looking for an explicit representation of this posterior probability in our SORN model (as suggested by [47]). Instead, we follow the idea of the sampling hypothesis [45, 48] and interpret the binary decision of the SORN for “A” or “B” as a one-shot sample from this posterior probability distribution *p*(A|*f*_A_) upon the one-shot presentation of the stimulus *s*. Upon every new presentation of the same stimulus *s* we expect to get a new sample from the same posterior. Thus we can experimentally measure the (binary) empirical distribution of the network’s stochastic answers and compare those results with the posterior according to the probabilistic model above.

**Fig 7:**
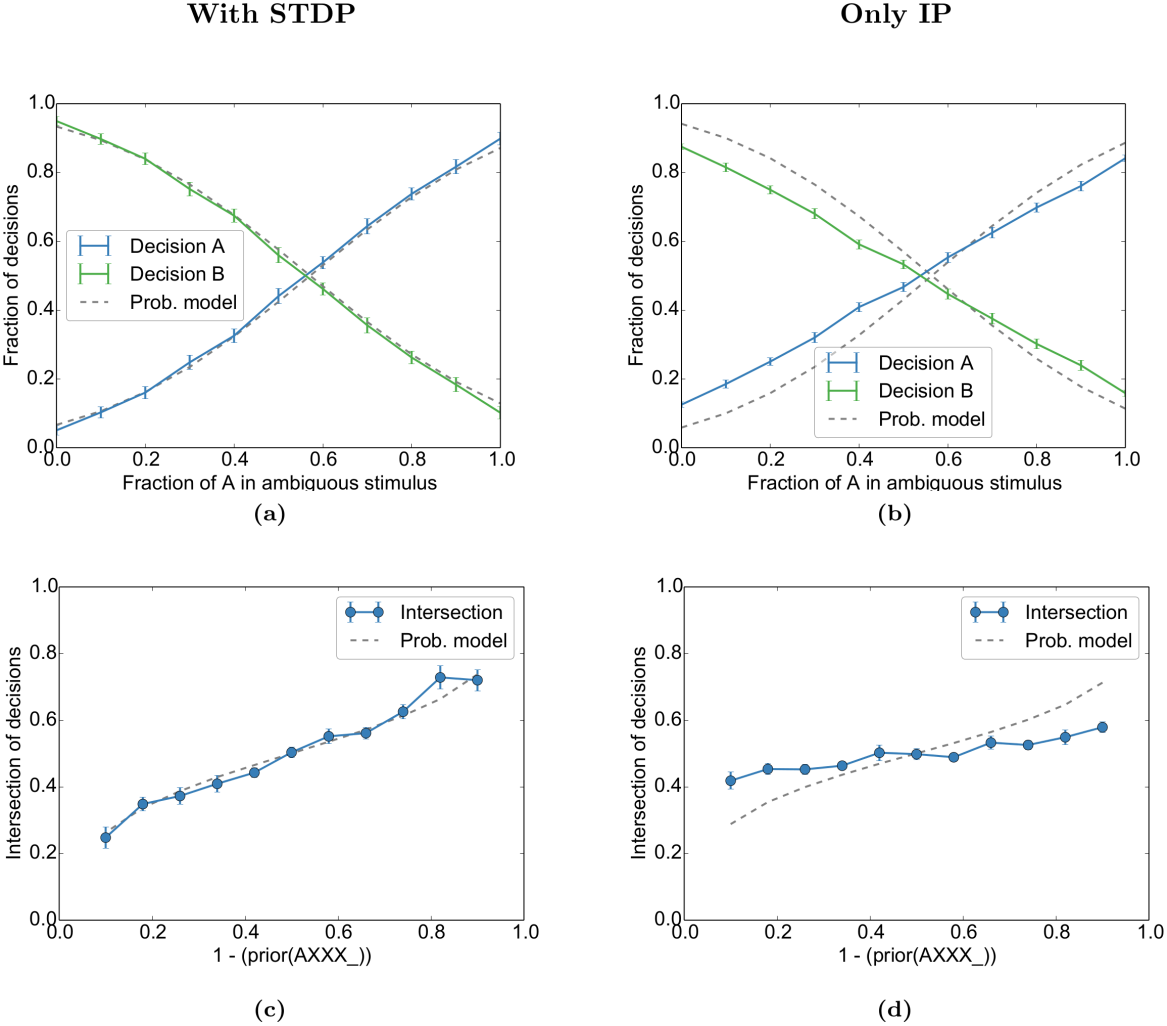
Prior and ambiguous information is combined in network decisions. (a) As in Fig 6, the network self-organizes during repeated presentations of the sequences “AXXX_ _ _ …” (33%) and “BXXX_ _ _ …” (67%) with blank intervals in between. In the test phase, an ambiguous mix of cue “A” and cue “B” is presented (“A/BXXX_ _ _ …”) and the network decides at the first “_” with a linear classifier if either A or B was the start of the sequence. The fraction of decisions for “A” or “B” at the first blank state “_” approximates the integration of cue likelihood and prior stimulus probability as expected from the probabilistic model (grey dashed line) for the given prior (*p*(A) = 0.33). The probabilistic model was fitted by grid search over its two parameters to the parameters that had the smallest accumulated error over all priors. (b) For the “Only IP” condition, STDP was deactivated during the self-organization phase and a new probabilistic model was fitted. (c, d) The intersections of the decisions for different priors for our simulations (blue line) and the probabilistic model (grey dashed). The performance was evaluated for the same simulation as Figs 5 and 6. Error bars represent SEM over 20 independent realizations.

The experimentally observed fractions of decisions for either “A” or “B” (blue and green lines in Fig 7a) approximate the Bayesian posterior *p*(A|*f*_A_) and *p*(B|*f*_A_) (grey dashed lines) for different ambiguity fractions *f*_A_ after convergence with learned priors of *p*(A) = 0.33 and *p*(B) = 0.67. We compare the results with an identical setting in which the STDP was turned off, but IP left on, to disentangle the respective effects (Fig 7b). The parameters of the probabilistic model were independently fitted to the simulation without STDP. In order to get a compact overview on the inference behaviour of the model across different values of the priors we especially observe the point of intersection of the blue and the green line and ask, which is the mixture fraction *f*_A_ of the two stimuli “A” and “B” that is necessary in order to counterbalance the prior such that the probability of the model’s response for “A” and “B” are both 50%. We plot these neutral values of *f*_A_ across different priors from *p*(A) = 10% to *p*(A) = 90%, both for the full model (blue line in Fig 7c) and for the case where STDP was turned off during the learning phase, leaving only IP for the adaptation of the excitabilities (Fig 7d). We compare them to the corresponding solutions of *p*(A|*f*_A_) = 0.5 from the probabilistic model (grey dashed line). Note that we always train the readout classifiers for the classes “A” and “B” with an equal number of positive and negative examples regardless of the prior, in order to avoid a trivial classification bias.

The result clearly reflects the correct incorporation of the prior—learned by self-organisation with STDP—into the activity upon an ambiguous stimulus. The homeostatic adaptation alone (IP only) only accounts for a small part of this adaptation capability, but provides a bias towards the right behaviour.

Two factors interact in the network in order to learn the above likelihoods and the model. First, firing thresholds of the input units are regulated by intrinsic plasticity. This entails that the “A”-neurons will not fire every time “A” is presented because they might have fired too frequently in the past. Second, input units (and neurons further downstream) for stimulus “A” can be active when the network is presented with stimulus “B” due to ongoing spontaneous activity. These two mechanisms ensure that ambiguous activations of the input populations are already present during the self-organization and learning phase. During the test-phase with ambiguous stimuli, the ongoing spontaneous activity will again lead to suppression or enhancement of “A” or “B” by either adding additional activity or suppressing activity. This can happen either directly through enhanced ongoing excitation or inhibition or indirectly through previous over- or under-activity leading to higher or lower thresholds. This effect of spontaneous activity on network decisions is the reason the decisions can be predicted from spontaneous activity in Fig 6.

Together, these results demonstrate that the network not only captures the experimentally observed interactions of spontaneous and evoked activity, it also integrates prior and ambiguous information while doing so. While the resulting behaviour looks sampling-like it is not clear if this model can learn more complex probability distributions. However, the ability of SORN models to solve much more complex sequence learning tasks is well documented (see, e.g., [37, 38] and Discussion for details).

### Network analysis

Given all these data, the question arises how the underlying mechanisms interact to give rise to this variety of features. To better understand the dynamics of the neurons, we determine the conditional probability for neuron *x*_*i*_ to spike given that neuron *x*_*j*_ spiked at the prior time step when no input is presented. This will elucidate both how the excitatory connectivity affects the network dynamics and how the network dynamics affect the connectivity via STDP.

#### Single-unit analysis

**Fig 8:**
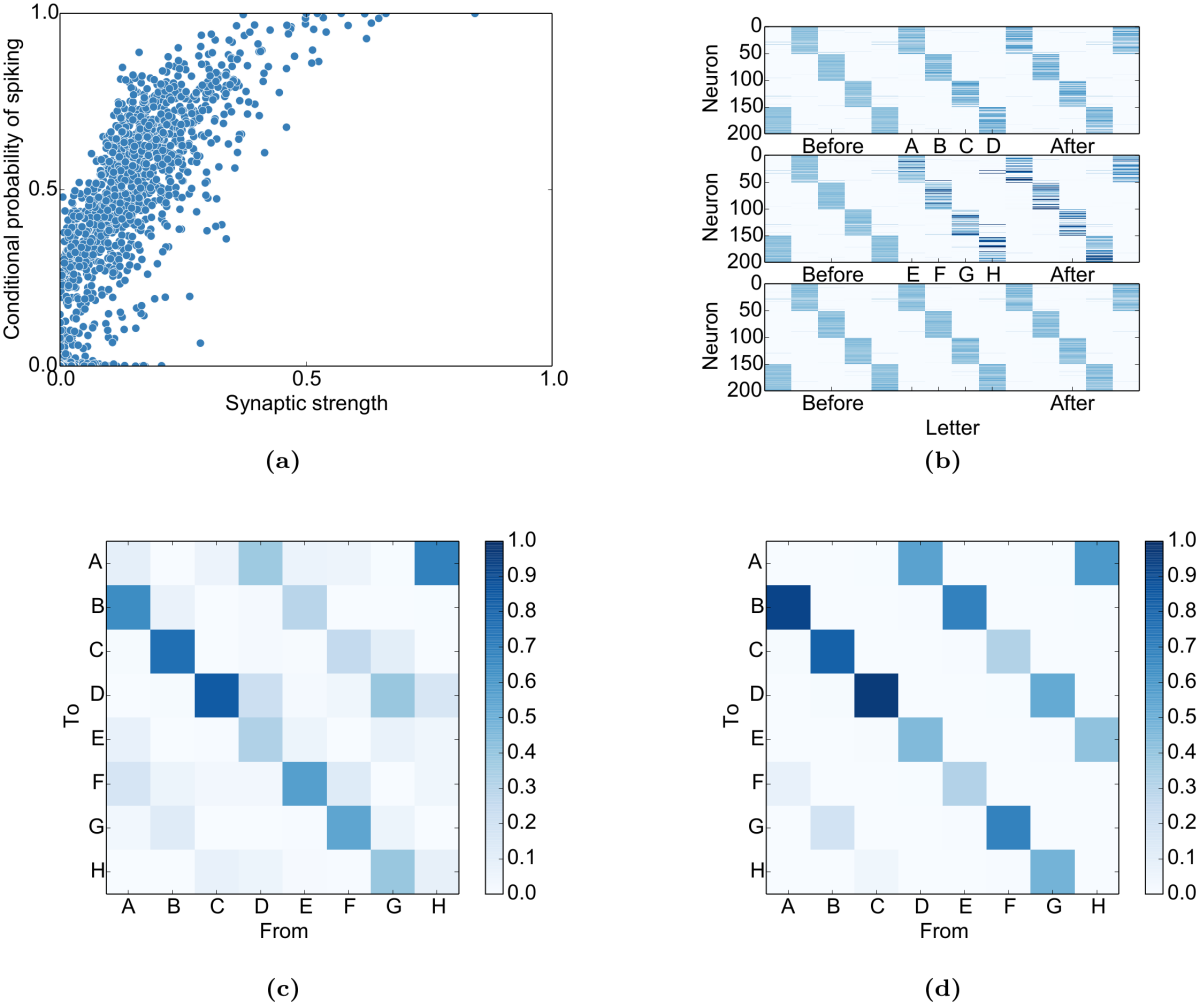
Self-organization imprints stimulus properties in the network. (a) The conditional probability of *x*_*i*_ spiking given that *x*_*j*_ just spiked (*p*(*x*_*i*_(*t* + 1) = 1|*x*_*j*_(*t*) = 1)) is roughly proportional to their synaptic connection $W_{ij}^{EE}$ except for saturation effects for very large weights. (b) The firing probabilities of each neuron relative to stimulus onset. The network develops sequential activity patterns during the presentation of both sequences (top and middle) and during spontaneous activity (aligned to spontaneous states corresponding to “C”, bottom). Neurons were sorted according to their maximal firing probability relative to the sequence “ABCD”. (c) The prediction of transition probabilities during spontaneous activity from the singular value decomposition of **W** ^*EE*^. (d) The actual transition probabilities during spontaneous activity show intermediate switching between the two sequences probably due to variability introduced by intrinsic plasticity (see Discussion for details).

We can see in Fig 8a that the conditional probability of neuron *x*_*i*_ spiking given that neuron *x*_*j*_ spiked at the previous time step (*p*(*x*_*i*_(*t* + 1) = 1|*x*_*j*_(*t*) = 1)) grows roughly linearly with the synaptic weight $W_{ij}^{EE}$ between both neurons except for saturation effects: $p\left(x_{i}\left(t+1\right)=1|x_{j}\left(t\right)=1\right)\approx \kappa W_{ij}^{EE}$. This relation, as simple as it might seem, immediately breaks down if IP or SN are deactivated (S6 Fig): While the general and intuitive trend persists that high weights imply higher conditional firing probabilities, the linear relation vanishes. The conditional probabilities in these figures were always computed for the spontaneous phase after self-organization to avoid effects from the input.

This relation between connection strength and firing probability interacts with STDP (but does not result from it, see S6 Fig): Given two reciprocal weights *W*_*ij*_ and *W*_*ji*_ with *W*_*ij*_ > *W*_*ji*_, the average weight update will be (given the target firing rate from the intrinsic plasticity, *H*_IP_)

$$\text{E}[\Delta W_{ij}^{EE}]\;=\;\eta_{\text{STDP}}(p(x_{i}(t+1)=1,x_{j}(t)=1)\;-\;p(x_{j}(t+1)=1,x_{i}(t)=1))\;$$ (1)

$$\begin{array}{cc} & \;=\;\eta_{\text{STDP}}(p(x_{i}\left(t+1\right)=1|x_{j}\left(t\right)=1)p\left(x_{j}\left(t\right)=1\right)-\\ & \;p(x_{j}\left(t+1\right)=1|x_{i}\left(t\right)=1)p\left(x_{i}\left(t\right)=1\right)) \end{array}$$ (2)

$$\begin{array}{cc} & \;\approx \;\eta_{\text{STDP}}\left(\kappa W_{ij}^{EE}H_{\text{IP}}-\kappa W_{ji}^{EE}H_{\text{IP}}\right) \end{array}$$ (3)

$$=\;\eta_{\text{STDP}}\left(\kappa H_{\text{IP}}(W_{ij}^{EE}-W_{ji}^{EE})\right)$$ (4)

>0. (5)

We observe that the expected weight update is directly proportional to the weight difference. In the likely case of the reciprocal weight being 0, it is directly proportional to the weight. This leads to the rich-get-richer behaviour of synaptic strengths as observed in a prevous version of the SORN [39]. Basically, a high weight increases the firing probability of the postsynaptic neuron upon presynaptic activation, which further increases the weight Eq (4), and so on. Another key factor for the rich-get-richer behaviour is synaptic normalization, as described in [39].

Apart from the skewed weight distribution, it eventually also leads to a sequential activation of neurons as observed in neocortex [28], in a previous version of the SORN [49], and in the current study (Fig 8b).

Next, we analyse the impact of input structure on the STDP dynamics. For this, we consider the case where an excitatory neuron *x*_*A*_ receives external input with the frequency *p*(*A*). Furthermore, we assume that this neuron projects to a second excitatory input-receiving neuron *x*_*B*_ with a very small weight, i.e. $W_{BA}^{EE}<\epsilon$, so that the impact of the weight on the firing probabilities can be neglected. Finally, we assume that there is no reverse projection from *x*_*B*_ to *x*_*A*_, i.e. *x*_*B*_(*t*) is assumed to be approximately independent from *x*_*A*_(*t* + 1). This neuron receives input from the next letter in the input word, “B”. We further assume that the stimuli are presented infrequently enough so that the intrinsic plasticity does not interfere with the activation of input-receiving neurons when the corresponding input is presented, i.e. *p*(*A*) ≪ *H*_IP_. This ensures that whenever *x*_*A*_ is activated by the input, *x*_*B*_ will be active in the subsequent time step with a probability close to 1. In this case, we have

$$\text{E}[\Delta W_{BA}^{EE}]\;=\;\eta_{\text{STDP}}(p(x_{B}(t+1)\;=\;1|x_{A}(t)\;=\;1)p(x_{A}(t)\;=\;1)\;-\;p(x_{A}(t+1)\;=\;1|x_{B}(t)\;=\;1)p(x_{B}(t)\;=\;1))$$ (6)

$$=\eta_{\text{STDP}}\;\left(\left(\frac{p(A)}{H_{\text{IP}}}\times 1+\frac{\left(H_{\text{IP}}-p(A)\right)}{H_{\text{IP}}}\times H_{\text{IP}}\right)H_{\text{IP}}-H_{\text{IP}}^{2}\right)$$ (7)

$$=\eta_{\text{STDP}}\;\left(p(A)-p(A)H_{\text{IP}}\right)$$ (8)

$$=\eta_{\text{STDP}}\;p(A)(1-H_{\text{IP}}).$$ (9)

The first term in Eq 7 corresponds to the conditional probability of firing when the firing of *x*_*A*_ is due to the stimulus, which will by assumption also lead to an activation of *x*_*B*_ with probability 1, or due to the chance event that a spontaneously driven spike in *x*_*A*_ is followed by a spontaneously driven spike in *x*_*B*_, which is *H*_IP_. These spontaneously driven spikes can either be due to recurrent activations from spontaneous activity in presynaptic units or due to the threshold of the unit being lowered below the current drive by intrinsic plasticity. Both probabilities are combined according to their relative occurrence: $\frac{p(A)}{H_{\text{IP}}}\times 1$ for the stimulus-driven case and ($\frac{\left(H_{\text{IP}}-p\left(A\right)\right)}{H_{\text{IP}}}\times H_{\text{IP}}$) for the recurrently driven one.

We observe that the expected weight change is directly proportional to *p*(*A*). As a result of this, if we consider two stimuli with different frequencies, the weights will grow stronger for the more frequent stimulus. In fact, they are directly proportional so that a stimulus that is twice as frequent will have a twice as strongly growing weight in the network. Taking into account the proportionality between the weight and the conditional firing probability and assuming initially random weights, this means that on average the more frequently presented stimulus during learning will also be associated with stronger weights and have a higher probability of recall. It is important to keep in mind that this specific case assumes that *x*_*A*_ receives direct input. Weights between pairs with only indirect input on *x*_*A*_ will be corrupted from unreliable activation of the presynaptic direct-input neurons of *x*_*A*_.

The above proportionality will break down if the weights become strong enough to have a strong impact on firing probabilities. This case will again result in rich-get-richer behaviour. This will lead to the overrepresentation of probabilities (“overlearning”) as observed in Fig 4. Nevertheless, the Figure also shows that despite this overrepresentation, the network does not completely abolish the more infrequent stimulus at convergence since the input still has an effect in driving the neuron and thereby the learning.

#### Population analysis

Having analysed the interactions of single neurons, it is important to know if these results generalize to the population as a whole. More specifically:

1. Can STDP imprint the sequential input structure in the recurrent excitatory connectivity?
2. Does the excitatory connectivity correspond to the observed transition probabilities of the network states when it is running without input?

We analyse these question by simplifying the excitatory network dynamics to a linear dynamical system:

$$x(t+1)\;=\;W^{EE}x(t).$$ (10)

We then apply singular-value decomposition (SVD) to the recurrent weight matrix:

$$W^{EE}\;=\;\;U\Sigma V^{T}.$$ (11)

Because **U** and **V** are orthonormal and **Σ** diagonal, we have

$$u_{i}\sigma_{i}\;=\;U\Sigma V^{T}v_{i}\;=\;W^{EE}v_{i}.$$ (12)

where **u**_*i*_ and **v**_*i*_ are column-vectors of **U** and **V** and *σ*_*i*_ is the corresponding singular value in **Σ**. By comparing Eqs (10) and (12), one can see that the vectors **v**_*i*_ and **u**_*i*_ *σ*_*i*_ define transitions similar to **x**(*t*) and **x**(*t* + 1). We therefore analyse the behaviour of learned connections by matching each vector **v**_*i*_ and **u**_*i*_ to their closest matching evoked state. This is done by taking the maximum of the dot product between the vector and the last 2500 evoked activity states of the training phase. Thereby we get from transitions between vectors to transitions between input letters. By scaling each transition by its singular value *σ*_*i*_ and normalizing to 1, we can predict the transition probabilities.

In Fig 8c, the result of this analysis is applied to the sequence learning task. When compared to the actual transitions during spontaneous activity in Fig 8d, one can see that the SVD-analysis approximates the actual transition probabilities. This entails that STDP can indeed imprint the input structure in the weight matrix and that these probabilities are correctly represented during spontaneous activity. The corresponding results for the inference tasks can be found in S5 Fig. Scatter plots for the spontaneous and predicted transitions can be found in S7 Fig.

Taken together, these analyses explain why and how the network activity acquires the stimulation structure in Fig 3 and how the input priors can be both imprinted into the network connectivity (cp. Fig 4) and utilized during testing (cp. Fig 7).

## Discussion

We have shown that key properties of neural variability emerge in a simple deterministic network of recurrently connected spiking neurons that learns a predictive model of its sensory environment. These key properties include the decrease of trial-to-trial variability with stimulus onset [10], the outlining of evoked responses by spontaneous activity [12, 13], the adaptation of spontaneous activity towards average evoked activity over training [16] and the prediction of evoked activity and perceptual decisions on the basis of spontaneous activity [8, 20]. While we are not the first to model these effects, we are, to the best of our knowledge, the first to account for all these effects in unison and in such a simple model. For example, constrained balanced networks can capture the decline of the Fano factor [50, 51]. However, these models do not employ learning and therefore cannot account for the other properties of neural variability treated here (but see [52]).

We hypothesized that three properties of cortical circuits lie at the heart of the above phenomena: 1. Recurrent connectivity shapes the structure of spontaneous activity and determines the relationship between spontaneous and evoked activity patterns. 2. Neural plasticity is responsible for structuring recurrent connectivity such that spontaneous activity matches the statistics of evoked activity. In functional terms this corresponds to the network learning a predictive model of its sensory environment. 3. Homeostatic mechanisms keep spontaneous and evoked activity in a healthy dynamic regime where learning and inference are possible. To test whether these properties are indeed sufficient to reproduce the above phenomena, we chose a bottom-up modelling approach.

We implemented a minimal network model embodying these properties and found that the network does reproduce the key experimental observations on neural variability and spontaneous activity. The model we chose is an instance of the family of self-organizing recurrent neural network (SORN) models. It is important to highlight that the network that formed the basis for the present work had been developed in a completely different context and with a very different goal. The SORN was originally introduced by [37] who showed that it is superior to a conventional reservoir computing approach when learning sequences with high-order Markovian structure. This was shown to be due to the unsupervised learning of the input structure with the same combination of plasticity rules used in the present work. Recently, [39] showed that a very similar network reproduces biological data on synaptic weight statistics and fluctuations. Their model accounted for both the lognormal-like distribution of excitatory-excitatory synaptic connection strengths (Fig 1f) and the fluctuations of individual synaptic efficacies over time. More recently, a different group independently validated this model on a grammar-learning task and found that the SORN displayed behaviour similar to humans learning the same grammars [38].

The specific implementation of SORN that we have used is only slightly different from the original version [37] in two ways: First, we made use of a presynaptic normalization in this study. This means that not only is the sum of incoming connection weights to a neuron normalized, but also the sum of all outgoing connection strengths is normalized. We find that this makes the model somewhat more robust. Mathematically speaking, normalizing the connections from both sides yields a weight matrix that is a linear combination of permutation matrices. This implies that in a linear dynamical system, the total activity would always be preserved, which gives an intuition as to why the nonlinear SORN model is more robust with this mechanism. While presynaptic normalization is well-known in the computational literature (see, e.g., [53]), there seem to be few biological experiments addressing the issue. We are aware of early studies on the “conservation of axonal arbor”[54, 55] suggesting that presynaptic normalization may be possible. Second, instead of using the same target firing rate for all neurons for the intrinsic plasticity, we introduced slightly different firing rates for each neuron. As for the presynaptic normalization, we found that this increases the robustness of the network dynamics: because each neuron has to fire with a slightly different rate, the emergence of repetitive activity patterns is discouraged and the network activity becomes more variable.

The simplicity and abstract nature of the SORN model allowed us to reproduce data on the interaction between spontaneous and evoked activity obtained from multi-electrode recordings over optical imaging to even fMRI. This generality allows us to predict that the phenomena that so far have only been reported for slowly-varying imaging data (e.g [8, 12, 20]) are also present on the spiking level. We predict that the population spiking activity prior to stimulus onset can be used to predict evoked spiking activity and perceptual decisions. We also predict that spontaneous spike patterns are shaped by learning and reflect the presentation probabilities of associated stimuli. Another concrete prediction is that the Fano factor decreases more strongly at stimulus onset for stimuli that have a higher probability of occurrence (Fig 5). In line with this prediction, a recent study has demonstrated that the Fano Factor decreases most for the preferred direction when analysing MT responses to moving gratings [44].

A less straight-forward effect is the overlearning found in Fig 4 and analysed at the end of the Results: due to the influence of recurrent reactivation of already learnt sequences on the learning process, very frequent stimuli will tend to have a reinforcing influence during learning and thereby become overrepresented in the network. This in turn suppresses infrequent stimuli. This simple interaction seems to be an inevitable feature of learning in recurrent networks. We speculate that sequence learning *in vivo* (as e.g. in [31]) also may not stop at the exact relative probability but tend to overrepresent very frequent stimuli.

Obviously, the model also has a number of limitations. The abstract nature of the model (memory-less threshold units operating in discrete time) makes it difficult to perform detailed comparisons to specific data sets. For example, while we showed that the spontaneous activity obtained in the model is highly structured and influences the evoked activity, we did not, say, attempt to reproduce the exact time course of the measured BOLD activity in the fusiform-face-area. Also, while there is evidence for the model’s plasticity mechanisms in neocortex and hippocampus (see Methods), we find that mainly unidirectional connections develop in the model due to the asymmetric STDP rule. This seems to be at odds with data on above-chance bidirectional connections (e.g. [56], but see [57]).

Another critical point are the Fano factor values we observe in the model. While [50, 51] succeeded in capturing the effect that most recordings in [10] show Fano factors above 1, we only observed Fano factors around 1 in the model. One possible explanation for this is suggested by the recent double-Poissonian model proposed in [40]. They demonstrated that the additional variability can be accounted for by a second, slowly varying factor such as the attentional state or levels of neuromodulation. Another study recently suggested that recurrent input not only from the local population but also from more distant brain areas leads to increased variability [22]. Since such processes are missing in our model, we also do not observe this additional variability.

### Relation to sampling theories

We chose a bottom-up modelling approach to address the question of the origin of neural variability and spontaneous activity. Ultimately, our goal is to reconcile such an approach with top-down functional approaches. Several features of neural variability and spontaneous activity have an elegant interpretation in terms of sampling theories of perceptual inference [45, 48]. In such theories, neural activity is interpreted as samples from a posterior probability distribution over the states of certain variables of interest given a sensory input. Correspondingly, spontaneous activity in the absence of sensory input is interpreted as samples from a prior probability distribution over these variables. This view elegantly captures several of the key experimental observations. For example, in this view the decrease in response variability after stimulus onset simply reflects that the posterior distribution over properties of the stimulus once it has been observed will be “narrower” compared to the prior distribution before stimulus presentation. The same holds for the outlining of evoked responses by spontaneous activity.

We showed that a fully deterministic SORN model can exhibit certain features expected from sampling-based inference (see, e.g., [45]). It may appear surprising that the behaviour of a fully deterministic network exhibits certain features of a probabilistic sampling strategy, but recent work has shown that Boltzmann Machines can be approximated by a sufficiently chaotic system [58]. It is well-known that such chaos can arise in deterministic neural networks similar to the SORN, for example by balanced excitation and inhibition [59]. Previous work with SORN models has also shown that they can exhibit dynamics close to the critical transition point between ordered and chaotic dynamics [60]. In principle, therefore, it cannot be ruled out that the SORN can be related to network implementations of sampling algorithms.

Hence, it is insightful to contrast our study with top-down modeling studies that tried to construct neural implementations of the sampling theory. Most prominently, the authors of [61] showed in a rigorous analysis that in a feed-forward-like structure with winner-take-all circuits, STDP can be used to approximate a sampling-version of expectation maximization and thereby learn a generative model of the input. Similarly, [62] demonstrated that Markov chain Monte Carlo sampling can be approximated with symmetrically connected spiking neurons. A different group showed that spatio-temporal patterns can be entrained in a network with an artificial importance-sampling rule [63]. A more biological approximation of this again resembles STDP. Finally, the model in [61] was recently extended to demonstrate that sequence learning in a Hidden Markov Model can again be approximated by using only a local STDP rule [64]. Therefore, recent top-down modelling studies demonstrate that STDP is a good candidate to learn appropriate weights to represent statistics of the input (see also [65]). This suggests that it may be possible to reconcile the bottom-up model presented here with top-down approaches for probabilistic inference by sampling, leading to a more complete understanding of neural variability and spontaneous activity. In this context it is worth pointing out that the top-down models discussed above make heavy use of intrinsic noise to achieve these sampling-effects (but see [66]). Our results, together with [58], suggest that this may not be necessary.

### How noisy is the brain?

In fact, our work casts some doubt on the heavy use of noise in other network models where it is used to stabilize networks in irregular regimes or to avoid oscillations or epileptic-like behaviour. This practice is usually justified by referring to data on neural variability in the cortex. Here we demonstrated that key findings on neural variability can be accounted for by a completely deterministic network learning a model of its sensory environment. Additionally, there is experimental evidence suggesting that action potential generation is highly deterministic and synaptic transmission becomes essentially deterministic as long as experiments are performed under realistic conditions [3–5]. Therefore, we propose that the common practice to make heavy use of noise in neural simulations should not be taken as the gold standard. Nevertheless, we also tested the robustness of our results to noise by adding Gaussian white noise with zero mean on the excitatory units and tracking the amount of spikes added and suppressed by the noise. We found that setting the variance of the noise to disturb up to 10% of the activity does not relevantly alter the results presented here.

So just how noisy is the brain? What do our results imply for the amount of noise we should expect to find in the brain? And what processes deserve to be called “noise” in the first place? From an information theoretic perspective, a natural assumption when theorizing about sensory processing is that the brain is trying to maximize the conditional mutual information MI(W;R|S) between sequences of states of the outside world *W* (or relevant parts thereof) and the internal representation *R* of this state sequence in terms of spatio-temporal cortical activity patterns, given the animal’s current brain state *S*. Conditioning on *S* expresses that the spatio-temporal activity patterns elicited by sensory inputs will depend on the current brain state of the animal, which is the product of its life-long experience and recent history of interaction with its environment. In any case, this mutual information can be decomposed as MI(W;R|S)=H(R|S)-H(R|W,S). Thus, in order to maximize MI(W;R|S) the brain should try to maximize the entropy of its internal representation of the world *H*(*R*|*S*) while at the same time trying to minimize the conditional entropy of this representation given the future sequence of states of the world and its current brain state *H*(*R*|*W*, *S*). Maximizing the first term also implies maximizing a lower bound of *H*(*R*), which means that the brain should make use of a maximally rich and diverse set of responses that, to an observer who does not have access to the true state of the world and the full brain state, should look like noise. In fact, the more efficient the code, the more the brain activity should look like noise. We speculate that this is the true reason why the activities of neurons are often so well-described by simple stochastic models. Minimizing the second term means that the ideal encoding should be deterministic, i.e., *R* is a deterministic function of *W* and *S*, because then *H*(*R*|*W*, *S*) reaches its smallest possible value of zero. Put simply, the best coding mechanism will have as little intrinsic noise as possible. Note that this does not imply that the neural response to a repeated stimulus will necessarily be the same (or even similar!). It only implies that it is a deterministic function of the current sensory input and the animal’s constantly evolving brain state. On the surface, the great response variability of neurons in sensory cortices seems to be at odds with the idea of a deterministic encoding. However, our results show that there exists a simple way of learning a fully deterministic coding scheme that is consistent with the key features of neural response variability observed in the brain. This raises the possibility that the brain may be using a highly efficient deterministic coding strategy and that for many years neuroscientists have mistaken this deterministic neural code for an inefficient and noisy one.

## Methods

### Model

Our model is based on the self-organizing recurrent neural network (SORN) [37]. Pilot studies related to the work presented here have been presented at a conference [67]. For more detailed information and a validation of the results, the code is available on github: https://github.com/chrhartm/SORN.

#### Network setup

The network consists of a population of *N* ^*E*^ = 200 excitatory and *N* ^*I*^ = 0.2 × *N* ^*E*^ = 40 inhibitory McCulloch & Pitts threshold neurons [68]. The connections between the neurons are described by weight matrices where, for example, $W_{ij}^{EI}$ is the connection from the *j* ^th^ inhibitory neuron to the *i* ^th^ excitatory neuron. We model the excitatory to excitatory connections as a sparse matrix with a directed connection probability of *p* ^*EE*^ = 0.1 and no excitatory autapses $W_{ii}^{EE}$. **W** ^*EI*^ as well as **W** ^*IE*^ are dense connection matrices. We do not model connections within the inhibitory population. All weights are randomly drawn from the interval [0,1] and then normalized by the synaptic normalization described below.

The input to the network is modelled as a series of binary vectors **u**(*t*) where at each time step *t* during input presentation all units are zero except for one. For better readability we assign an arbitrary letter to each such state when describing different input sequences later on. The letter “_” corresponds to presenting no input. Each input unit *u*_*i*_ projects to *N* ^*U*^ = 10 excitatory neurons with the constant weight *w* ^*in*^ = 0.5. These randomly selected and possibly overlapping projections are represented by the input weight matrix **W** ^*EU*^.

At each discrete time step *t*, these variables contribute to the binary excitatory state **x**(*t*) ∈ {0, 1}^*N*^*E*^^ and inhibitory state **y**(*t*) ∈ {0, 1}^*N*^*I*^^ as follows:

$$x(t+1)=\Theta \left(W^{EE}(t)x(t)-W^{EI}y(t)+W^{EU}u(t)-T^{E}(t)\right)$$ (13)

$$y(t+1)=\Theta \left(W^{IE}x(t+1)+W^{IU}u(t)-T^{I}\right).$$ (14)

Here, Θ(**a**) is the element-wise heaviside step function, which maps activations **a** to binary spikes if the activation is positive. **T** ^*E*^(*t*) and **T** ^*I*^ are the thresholds of all neurons and are crucial in regulating the spiking. They are evenly spaced in the interval (0, 0.5) for the excitatory thresholds and $\left(0,T_{max}^{i}\right)$ for the inhibitory ones.

$T_{max}^{i}$ directly influences the amount of inhibition. $T_{max}^{i}$ presents a trade-off between fine-grained inhibition and a broad dynamic range: for a low $T_{max}^{i}$, the additional excitation needed to activate one more inhibitory neuron is small, leading to a more fine-grained response to excitatory fluctuations, which is in general favourable because it helps to avoid pathological behaviour. However, a low $T_{max}^{i}$ sets a low bound on the maximal amount of inhibition: it is easier to activate all inhibitory neurons and thereby saturate the possible inhibitory response. Therefore, in cases with high fluctuations in excitation, such as trial-like settings with a high rate increase at stimulus onset, a high $T_{max}^{i}$ is necessary. Please note that the average amount of inhibition will have no effect on the average amount of excitation since the average excitatory activity in the network is regulated by the intrinsic plasticity. $T_{max}^{i}$ is set to $T_{max}^{i}=0.35$ for the first tasks and to $T_{max}^{i}=1$ for the inference task with its trial structure. If $T_{max}^{i}$ is set to 1, excitation and inhibition are balanced: For a given fraction of excitatory activity $\bar{x}=\frac{1}{N^{E}}\sum_{i=0}^{N^{E}}x_{i}\left(t\right)$, each inhibitory neuron receives on average $1\times \bar{x}$ excitatory input because synaptic normalization ensures that the summed excitatory incoming weight is 1. This in turn activates the fraction $\bar{y}=\frac{1}{N^{I}}\sum_{i=0}^{N^{I}}y_{i}\left(t\right)\approx 1\times \bar{x}$ of inhibitory neurons, because the thresholds of the inhibitory units are uniformly distributed in the interval (0, 1) by assumption. It is important to note that at no point any noise is added to the network. The only external variability is the random alternation of input words, as described in the Results.

The excitatory thresholds as well as **W** ^*EE*^(*t*) are adapted over time by three plasticity mechanisms. The state **x**(0) is randomly initialized with probabilities **T** ^*E*^(0) and **y**(0) is initially set to 0.

#### Plasticity mechanisms

The network employs three different kinds of plasticity: Spike-timing dependent plasticity (STDP) [69–71] extracts structure from the input by shaping the weights within the excitatory population. This is counterbalanced by two forms of homeostatic plasticity: intrinsic plasticity (IP) [72, 73] and synaptic normalization (SN) [32, 33, 74]. All these mechanisms are known to co-occur in the hippocampus and neocortex.

STDP strengthens the connection $W_{ij}^{EE}$ from unit *j* to unit *i* whenever a spike in *i* directly follows a spike in *j* (i.e. *j* helped to trigger *i*) and is weakened whenever a spike in *i* precedes a spike in *j*. This results in the following update equation (with *η*_STDP_ = 0.001):

$$\begin{array}{c} \Delta W^{EE}\left(t+1\right)=\eta_{\text{STDP}}\left(x\left(t+1\right)x{\left(t\right)}^{T}-x\left(t\right)x{\left(t+1\right)}^{T}\right). \end{array}$$ (15)

The authors of [33] showed in an electron microscopy study that the summed synaptic area per *μm* of dendrite is similar before and after long-term potentiation while the synaptic area per synapse increases and the number of synapses per *μm* decreases. This indicates that plasticity redistributes weights to avoid uncontrolled growth. This is modelled by normalizing all incoming connections of a neuron to 1—a process called synaptic normalization (SN). In addition to this postsynaptic normalization, there is also evidence for presynaptic normalization or “conservation of axonal arbor”[54, 55]. These studies suggest that all outgoing connections are redistributed to achieve a constant total weight. This is modelled by normalizing all outgoing connections of a neuron to 1. Together, these mechanisms result in the following iterative weight update:

$$\begin{array}{c} W_{ij}^{EE}\left(t+1\right)\leftarrow 0.9\times W_{ij}^{EE}\left(t+1\right)+0.1\times \frac{W_{ij}^{EE}}{0.5\times \sum_{k=0}^{N^{E}}W_{ik}^{EE}\left(t+1\right)+0.5\times \sum_{k=0}^{N^{E}}W_{kj}^{EE}\left(t+1\right)}. \end{array}$$ (16)

At the same time, there are a variety of regulatory mechanisms that control neural firing at different time scales such as absolute and relative refractory periods, spike rate adaptation and intrinsic plasticity [32, 73]. Here, we abstract from those by using a simple homeostatic regulation of the spike threshold at a single time scale (*η*_IP_ = 0.001). This intrinsic plasticity (IP) rule regulates the individual thresholds so that on average, the excitatory neurons will fire according to their target rates **H**_IP_:

$$\begin{array}{c} T^{E}\left(t+1\right)=T^{E}\left(t\right)+\eta_{\text{IP}}\left(x\left(t\right)-H_{\text{IP}}\right). \end{array}$$ (17)

The target rates are uniformly drawn from the interval (*H*_IP_ − *ϵ*_IP_, *H*_IP_ + *ϵ*_IP_) = (0.1 − 0.01, 0.1 + 0.01). The resulting diversity in firing rates helps to prevent pathological network activity.

The inhibitory connections are scaled during the initialization phase so that the sum of excitatory weights received by each inhibitory unit and the sum of inhibitory weights received by each excitatory unit is 1.

The STDP and IP rules are only operating on the excitatory neurons for two reasons. First, by restricting plasticity to only the excitatory population, the model becomes simpler and thereby easier to understand and interpret. Second, there are fewer data on plasticity in inhibitory neurons (but see [39]).

#### Stimulation paradigm

The stimulation paradigm is very similar across all tasks and can be divided into three phases:

In the *self-organization phase*, the network is stimulated with input patterns or sequences for *T*_*plastic*_ = 50000 steps. During this time, all plasticity mechanisms are active and the network can self-organize in the presence of the given input. We represent the input vectors by letters. For example, the stimulation paradigm can then be a random alternation of the words “ABCD” and “EFGH”. After this, STDP is switched off so that the properties of the learnt connections can be studied without interference from continued changes to the weights. This is done during a training and testing phase.

In the *training phase*, the stimuli are kept identical to observe the properties of the network under the self-organization conditions and training appropriate readouts for *T*_train_ = 20000 steps. For the model of the sequence prediction of [31], we do not show stimuli in this phase corresponding to the “sleep”.

Then, during the *testing phase*, we either observe the spontaneous activity while the network is not stimulated, or we test our readout on data generated by stimulating the network with appropriate stimuli for *T*_test_ = 50000 steps.

For these phases, the intrinsic plasticity is still active to ensure stable average activity. After each phase, the activity state is shuffled in accordance with [75]. To simulate the trial-like structure of the inference task, we chose to have blank periods between each stimulus presentation. The length of these periods was drawn from a uniform distribution over the interval [10, 15] time steps. For the model of the sequence learning study (Fig 2), we used a fixed delay of 10 time steps because the experiment only had fixed inter-trial delays [31].

### Analysis methods

In this study, we compare the behaviour of our model to a variety of experimentally reported results. While our network is operating on a timescale of roughly 25ms (the width of a typical STDP-window) and on the spiking level with rates around 4Hz, the experimental data are fMRI-BOLD signals, optical imaging data and multi-unit activity spike trains and have effects on time scales that range between milliseconds and seconds. Comparing these partly very different data can therefore sometimes be only done on an abstract and qualitative level. In the following section, we outline how each comparison was performed.

#### Principal component analysis and multidimensional scaling

For the principal component analysis (PCA) of the spontaneous and evoked states, we did a PCA of the last 2500 steps of the training phase. We then projected these states in the subspace defined by the first three components of the PCA and also projected the same amount of spontaneous activity states in the same subspace. The spontaneous states were taken from the end of the testing phase to avoid artifacts due to the re-adaptation of the thresholds after cutting the input.

Multidimensional scaling (MDS) was performed as closely as possible to the method used in [13]. As in that paper, we used 150 points of spontaneous, shuffled spontaneous and evoked samples. Further following the paper, we shuffled the spontaneous activity of each neuron individually over time so that neurons kept their rate but lost their timing. The samples were chosen randomly from the end of the training and testing phase. We did not subsample our neurons to the 45 that they recorded from because the distances between individual activity patterns become too similar in that case. This did not happen for their study because they used rates instead of spikes. To account for the fact that [13] only showed a subset of all the stimuli that the network was exposed to during its development we also only used a randomly chosen subset (5 letters) of the stimuli that we presented in the self-organization phase. We used the same Matlab function for MDS as the one used in the original paper (with Kruskal’s normalized stress1 criterion).

#### KL-divergence of spontaneous and evoked activity

To replicate the results of [16], we recorded spontaneous and evoked activity for the same number of time steps (750.000) and randomly subsampled 16 units from our network. Please note that due to an exponential increase in possible patterns with the number of units, more units would have soon become infeasible to analyse with conventional methods. We also excluded the first 5000 steps of each phase to account for adaptation after changes in the stimulation paradigm. To compute the KL-divergence we first have to estimate the probabilitiy *p*(*x*) for each pattern *x* from the set of the 2^16^ possible patterns *X*. In order to do so, we created a bin for each pattern and simply counted the occurrence of each pattern. Additionally, we started with a non-informative prior by assuming that each pattern *x* ∈ *X* was already observed once. This initial prior is necessary since KL-divergence is only defined for non-zero probabilities. After normalizing, we then computed the KL-divergence between evoked and spontaneous activity according to

$$D(\text{evoked}||\text{spont})\;=\text{ KL}(P^{\text{evoked}},P^{\text{spont}})$$ (18)

$$=\;\sum_{x\in X}P^{\text{evoked}}(x)\times \text{log}\frac{P^{\text{evoked}}(x)}{P^{\text{spont}}(x)}.$$ (19)

#### Pattern analysis

Evoked and spontaneous activity patterns may rarely be exactly equal. In order to relate spontaneous activity patterns to evoked activity patterns, we use the following method. We assigned to each spontaneous state the letter corresponding to the best-matching evoked activity state. If, for example, **x**(*t*) had the smallest Hamming distance to an evoked state when the letter “A” was shown, then **x**(*t*) was labelled as corresponding to input letter “A”. To avoid biases, the collection of evoked and spontaneous states used for the comparison was always obtained from the end of the training and testing phase (the last 2500 steps). The data was further reduced by ignoring blank periods and ensuring that each letter was represented equally often in the data set.

#### Inference analysis

To quantify the network’s behaviour in the inference task, we trained output units based on the randomly alternating presentation of the stimuli “AXXX_ _ _ …” and “BXXX_ _ _ …”, where “A”, “B”, and “X” each refer to a subpopulation of excitatory neurons that are stimulated whenever the letter is presented. At the presentation of “_”, no neurons receive external inputs. These stimuli model a decision task where subjects were presented with the ambiguous face-vase stimulus and had to decide whether they perceived a face or a vase after a mask [20]. “A” and “B” represent the face or vase, the following “X”s are identical for both stimuli and thereby act as a mask and delay.

To read out the model decisions after the mask, we trained a linear readout in a supervised way with least-squares regression. We performed two regressions from all neurons at the step when “_” is presented to predict either “A” or “B” (based on the recurrently maintained information of “A” or “B” that should still be present in the network). In the test phase, when ambiguous mixtures of “A” and “B” are presented, the network decision is set to “A” when the readout for “A” is larger than for “B” and the other way round. The regression target was set to 0 for all other letters from the training data, since these should not correspond to a “sampling” of “A” or “B”. The regression was performed on 20000 steps of evoked activity while STDP is turned off. To avoid any biases, we took equal amounts of activity samples for each letter from the end of training.

In the test-phase, the ambiguous face-vase stimulus is modelled by stimulating the network with a mix of “A” and “B”. This is done by using $f_{A}\times N_{E}^{U}=f_{A}\times 10$ input units of “A” and $\left(1-f_{A}\right)\times N_{E}^{U}=\left(1-f_{A}\right)\times 10$ input units of “B”. Here, we assume that stimulus ambiguity can be modelled by the fraction of activated “A” units, *f*_A_. The specific units selected for the ambiguous stimulus are redrawn at every stimulus presentation. To have well-defined ambiguities, we ensure for this task that the input populations of “A” and “B” do not overlap. As in the original study, the delay between stimuli is random. We model this by randomly adding between 0 and 5 delay steps to the fixed delay of 10 steps during testing (see stimulation paradigm for details).

#### Probabilistic model

A detailed inspection of the input populations for the inputs “A” and “B” reveals the root of the stochastic behaviour of SORN: One input neuron, say the first neuron of the population *P*_A_ representing input “A”, may be stimulated, i.e. its input set to *w* ^*in*^ = 0.5, but still that very neuron does not fire in this time step due to a stronger inhibition it receives via the recurrent connections and/or a higher activation threshold due to previous activity. Obviously, the information that the neuron was stimulated by the input in this time step is lost. On the other side the neuron might get active due to the excitatory recurrent input from the network and/or a drop of its threshold due to long inactivity, no matter if there is an external stimulation or not. Also in this case the stimulus had no changing effect on the evolution of the future states of the network and thus that information is lost. In a little more complex but similar way we can reason about the propagation of the effect of this one input stimulus through the network over time until the delayed readout tries to identify if population *P*_A_ or population *P*_B_ was stimulated. From an information theoretic viewpoint we can formalize this uncertainty as a noisy binary channel, which is fully characterized by two parameters *θ*_0|0_ and *θ*_1|1_, or shorter *θ*_0_, *θ*_1_, denoting the probability that a non-stimulus (0) and a stimulus (1) is received correctly. The probabilities of a wrong transmission are implicitly defined as well, since *θ*_1|0_ = 1 − *θ*_0_ and *θ*_0|1_ = 1 − *θ*_1_.

Based on this channel perspective we can define a simple Naive Bayesian classifier that receives those noisy inputs for population *P*_A_ and *P*_B_, denoted by **a** = {*a*_1_, …, *a*_10_} and **b** = {*b*_1_, …, *b*_10_}, during both the learning and the testing phase, assuming *N* ^*U*^ = 10 input units as defined in the SORN description above. It is straightforward to derive the parameters of such a Naive Bayes analytically, as there are only two training cases: When the sequence “AXXX___…” is presented in the learning phase, all neurons that belong to the population *P*_A_ receive an input stimulation, whereas all neurons of the *P*_B_ population do not receive any stimulus. Thus, according to our channel model all *a*_*i*_ have a probability of *θ*_1_ of being 1 and all *b*_*i*_ have a probability of 1 − *θ*_0_ of being 1, i.e. *p*(*a*_*i*_ = 1|A) = *θ*_1_ and *p*(*b*_*i*_ = 1|A) = 1 − *θ*_0_. In the second case, when the “BXXX___…”-sequence is presented, the whole *P*_B_-population is stimulated and the *P*_A_-population is left alone, thus in our probabilistic model *p*(*b*_*i*_ = 1|B) = *θ*_1_ and *p*(*a*_*i*_ = 1|B) = 1 − *θ*_0_. The prior *p*(A) just reflects how often the “AXXX___…”-sequence is presented to the model during learning as compared to the “BXXX___…”-sequence. The conditional probabilities together with the prior *p*(A) and *p*(B) = 1 − *p*(A) fully define the Naive Bayes model up to the two free parameters *θ*_1_ and *θ*_0_. We will derive these parameters by fitting the probabilistic model to the experimentally derived results from the SORN network.

Given certain evidence vectors **a** and **b** we can derive the posterior probability of the Naive Bayes model according to Bayes’ rule as

$$p\left(\text{A}|a,b\right)=\frac{p\left(\text{A}\right)p\left(a,b|\text{A}\right)}{p\left(\text{A}\right)p\left(a,b|\text{A}\right)+p\left(\text{B}\right)p\left(a,b|\text{B}\right)}$$ (20)

where the likelihood *p*(**a**,**b**|A) can be expressed explicitly in terms of the above defined parameters

$$p(a,b|\text{A})=\prod_{i=1}^{10}p(a_{i}|\text{A})\prod_{i=1}^{10}p(b_{i}|\text{A})$$ (21)

$$\begin{array}{cc} & =\theta_{1}^{n_{a}}{\left(1-\theta_{1}\right)}^{10-n_{a}}{\left(1-\theta_{0}\right)}^{n_{b}}\theta_{0}^{10-n_{b}}, \end{array}$$ (22)

where $n_{a}=\sum_{i=1}^{10}a_{i}$ and $n_{b}=\sum_{i=1}^{10}b_{i}$ are the sums of active evidences received from population *P*_A_ and *P*_B_, respectively. Note that **a** and **b** and thus also *n*_*a*_ and *n*_*b*_ refer to the evidences as seen by the Naive Bayes classifier which result from the hypothetical noisy transmission channels of the external activations. The posterior in terms of *n*_*a*_ and *n*_*b*_ reads

$$p(\text{A}|n_{a},n_{b})=\frac{p(\text{A})\theta_{1}^{n_{a}}{\left(1-\theta_{1}\right)}^{10-n_{a}}{\left(1-\theta_{0}\right)}^{n_{b}}\theta_{0}^{10-n_{b}}}{p(\text{A})\theta_{1}^{n_{a}}{\left(1-\theta_{1}\right)}^{10-n_{a}}{\left(1-\theta_{0}\right)}^{n_{b}}\theta_{0}^{10-n_{b}}+p(\text{B}){\left(1-\theta_{0}\right)}^{n_{a}}\theta_{0}^{10-n_{a}}\theta_{1}^{n_{b}}{\left(1-\theta_{1}\right)}^{10-n_{b}}}$$ (23)

Note that the binomial factors that would appear in *p*(*n*_*a*_, *n*_*b*_|A) are reduced in the above fraction. In order to evaluate the whole probabilistic model we also have to evaluate the noisy channels. In our experiments we always stimulate a certain (random) fraction *f*_A_ of neurons of population *P*_A_ and a (random) fraction of (1 − *f*_A_) neurons of population *P*_B_. The resulting evidence vectors **a** and **b** are characterized by the respective distributions of *n*_*a*_ and *n*_*b*_:

$$n_{a}\sim B(10f_{{}_{\text{A}}},\theta_{1})+B(10-10f_{\text{A}},1-\theta_{0})$$ (24)

$$n_{b}\;\sim \;B(10-10f_{\text{A}},\theta_{1})+B(10f_{\text{A}},1-\theta_{0}),$$ (25)

where *B*(., .) is the binomial distribution. We obtain the resulting probability from the probabilistic model upon stimulation with a stimulus with ambiguity *f*_A_ by taking the expectation of the posterior Eq (23) over the possible realizations of *n*_*a*_ and *n*_*b*_, i.e.

$$p\left(\text{A}|f_{\text{A}}\right)={\langle p\left(\text{A}|n_{a},n_{b}\right)\rangle }_{n_{a},n_{b}}$$ (26)

This function still depends on *θ*_1_ and *θ*_0_. In order to determine those parameter values we consider the full experiment in the SORN model and evaluate the decision curves over *f*_A_ (Fig 7a) for all different values of the prior during training (10% to 90%). Every such curve is the result of an average of 20 realizations of the—stochastically created—SORN model. Then we find those two parameter values *θ*_1_ and *θ*_0_ that fit all those 9 curves the best, in the sense of the least sum of the mean squared deviations. For the sake of simplicity the optimization is carried out by an exhaustive grid search in the range of 0.05, …, 0.95 with step size 0.05. The result of the search delivered the values *θ*_1_ = 0.85 and *θ*_0_ = 0.45 for the full SORN and *θ*_1_ = 0.95 and *θ*_0_ = 0.3 for the “Only IP” condition.

#### Fano factor analysis

The Fano factor is defined as $\frac{\sigma^{2}}{\mu }$. When applied to spike trains, the windowed variance *σ* ^2^ and mean *μ* are taken over trials for each neuron, condition and time step. It is then interpreted as the variability of the data. For a Poisson process, which describes the irregular spiking of neurons in many cases quite well, the Fano factor is 1 due to the variance and mean being equal for this process.

We tried to keep our analysis of the Fano factor close to [10]. As in the original paper, the FFs were obtained by weighted regression between the spiking mean and variance over trials. To compute these two, we used a causal sliding window with a width of 5 time steps, i.e. the sum of the spikes in the past four time steps and the time step to be analysed. Together with the IP parameters, this entails a rate of 0.5 spikes per bin on average. This is comparable to the conditions of the original paper: most experiments have a rate on the order of $10\frac{\text{spikes}}{s}$ and a bin size of 50ms. One should note that due to the weighted regression and the averaging over many neurons, the resulting Fano Factor does not have to be identical to the ratio of the mean variance and the mean firing rate.

To control for an effect of the mean firing rate on the Fano factor, we also performed the “mean matching” analysis from [10]. We found that this method discards around two thirds of the data, comparable to the original study. Therefore, we only see it as a control and focussed on the real FF for the discussion.

#### Prediction of evoked activity and decisions

For our last comparisons, we aimed to show that the spontaneous activity in this network can be used both to predict the following evoked activity [8] and to predict the decision of the network as for example in [19, 20].

The former was done in a similar way as the inference: For each combination of stimulus condition *c* and time step Δ*t*_*a*_ after stimulus onset *t*_on_, we trained a linear readout that maps **x** ^*c*^(*t*_on_ − 1) to **x** ^*c*^(*t*_on_ − 1 + Δ*t*_*a*_). The match between the predicted evoked activity and the actual evoked activity **x** ^*c*^(*t*_on_ − 1 + Δ*t*_*a*_) was then calculated by taking the pearson correlation between both states. We allowed a bias term in the regression by appending a constant to each **x** ^*c*^(*t*_on_ − 1) vector. To asses the impact of the spontaneous activity on the prediction, we compared this performance to doing the same regression from shuffled spiking patterns over trials. By shuffling over trials, the statistics of the individual patterns persist, but their relation to the decision vanishes. Since this still includes the bias term, this control can capture average effects but has to deal with the same number of parameters as the original regression. Therefore, if the spontaneous activity prior to stimulus onset contains information about the following evoked activity, the prediction based on the spontaneous activity should be better, i.e. correlate more with the true activity, than the one based on the shuffled spike trains. Also, both predictions should overlap for very late states since the information from the spontaneous activity should be “washed out” by then.

To predict the decision of the network, we also used this regression approach. For this, we divided the *T*_test_ steps of network activity with decisions into two halves—one for training the readouts to predict the decision for “A” and “B” and one for testing its performance. For each step before stimulus onset and a given stimulus, an individual readout was trained. The prediction was then defined as the higher readout. We evaluate the quality of this prediction by comparing it to the actual decisions of the network and computing the agreement between both for the testing data. These actual decisions are usually biased towards one of the two alternatives, either due to the prior of the stimulus in the self-organization phase or due to the initial structure of the network. This bias can be exploited by the bias term in the readout mentioned above to get above-50% performance for the baseline comparison, where again trial-shuffled spiking data plus a bias term is used to predict decisions.

## Supporting Information

S1 Fig Basic properties of the network from a representative trial of the sequence learning task. (a) A sample of spikes after plasticity. (b) The inter-spike-interval (ISI) distribution of a randomly selected neuron during spontaneous activity is well-fitted by an exponential after accounting for the periodic structure of the task. (c) The distribution of coefficients of variation (CVs) of the ISIs is on average slightly smaller than for the random letters (cp. Fig 1). (d) The fraction of excitatory-to-excitatory connections initially converges to a stable fraction, then decreases again. The network behaviour does not change despite the transient decrease. (e) Individual weights fluctuate despite the global periods of convergence. (f) After self-organization, the binned distribution of excitatory-to-excitatory synaptic weights (dots) is well fit by a lognormal distribution (solid line).
(TIF)

S2 Fig Basic properties of the network from a representative trial of the inference task. (a) A sample of spikes during the test phase. Stimuli were presented in the shaded areas. (b) The inter-spike-interval (ISI) distribution of a randomly selected neuron during spontaneous activity reflects the trial-like structure during learning with pauses between activity bursts. (c) The distribution of coefficients of variation (CVs) of the ISIs clusters around one. (d) The fraction of excitatory-to-excitatory connections converges to a stable fraction. (e) Individual weights fluctuate despite the global convergence. (f) After self-organization, the binned distribution of excitatory-to-excitatory synaptic weights (dots) deviates from a lognormal distribution (solid line).
(TIF)

S3 Fig Sequences separate at higher principal components. In the simulation of Fig 3a, the network was stimulated with the words “ABCD” (67%) and “EFGH” (33%) and evoked (dots) and spontaneous (lines) activity were projected into the principal component space of the evoked activity. Here, we display the 4th, 9th, and 11th principal component (PC) from that simulation. In each dimension, one letter of the two sequences clearly separates. The less variability is explained by the PC (i.e. the higher the PC number), the earlier the letter occurs in the sequence. As argued in the text, this effect might be due to small differences at the beginning accumulating through the recurrent structure to larger differences towards the end of the word. As expected from the other results in Fig 3, the spontaneous activity follows the structure of the sequences. For example, it transitions from blue to green (“B” to “C”) or from gold to pink (“F” to “G”) but not from blue to pink or form gold to green. This causes the opposing triangles of spontaneous activity in the plot.
(TIF)

S4 Fig Mean-matched Fano factors. To control for effects of the mean, we computed the Fano factors with the mean-matching method proposed by [10]. This was done for both (a) the inference task and (b) the sequence learning task. As in the original paper, we averaged over all conditions. Therefore, we cannot distinguish between stimuli as we did in Fig 5. Both conditions used the same prior as in Figs 3 and 7. Please note that the sequence task does not have a trial structure but the network is continuously stimulated. Therefore, there is on average no rate change in raw activity at the onset of each stimulus. Error envelopes are SEM over 20 independent realizations.
(TIF)

S5 FigNetwork analysis for the inference task.(a) The conditional probability of spiking *p*(*x*_*i*_(*t* + 1) = 1|*x*_*j*_(*t*) = 1) is directly proportional to its synaptic weight $W_{ij}^{EE}$. (b) The firing probabilities of each neuron relative to stimulus onset. The network develops sequential activity patterns during the presentation of both sequences (top and middle). Neurons were sorted according to their maximal firing probability relative to the sequence “AXXX_ _ _ …”. (c) The prediction of transition probabilities during spontaneous activity from the singular value decomposition of **W** ^*EE*^. (d) The actual transition probabilities during spontaneous activity. Please note the prominent “_” to “_” transitions reflecting the blank periods during training.(TIF)

S6 Fig IP and SN are essential for healthy network dynamics. (a) Conditional firing probabilities from Fig 8a. (b) The original simulation without STDP demonstrates that STDP is not necessary for the linear relation between synapse strength and firing probability. Also, STDP leads to stronger weights. (c) The original simulation without IP shows that IP is essential for maintaining correct firing probabilities. (d) The same holds true for a simulation without synaptic normalization. Both (c) and (d) also show incorrectly learnt weight matrices and pathological network dynamics (see [37] for effects of excluding IP or SN on network dynamics).
(TIF)

S7 Fig Spontaneous transitions vs. SVD prediction. (a) The transition probabilities between letters during spontaneous activity and its estimates from singular value decomposition are plotted for the sequence learning task. The green line is the fitted linear regression. The individual transition probabilities are shown in Fig 8c and 8d. (b) The same plot for the data from the inference task (S5c and S5d Fig) shows a worse match. This is due to more spontaneous activity during training and the thereby less constrained excitatory weight matrix. This results both in smaller transition probabilities during spontaneous activity and a more ambiguous SVD analysis of the weight matrix.
(TIF)
