# Supplementary figures and images for: Where’s the Noise? Key Features of Spontaneous Activity and Neural Variability Arise through Learning in a Deterministic Network
Source: PLoS Comput Biol. 2015 Dec 29;11(12):e1004640. doi: 10.1371/journal.pcbi.1004640 (PMC4694925; doi:10.1371/journal.pcbi.1004640)

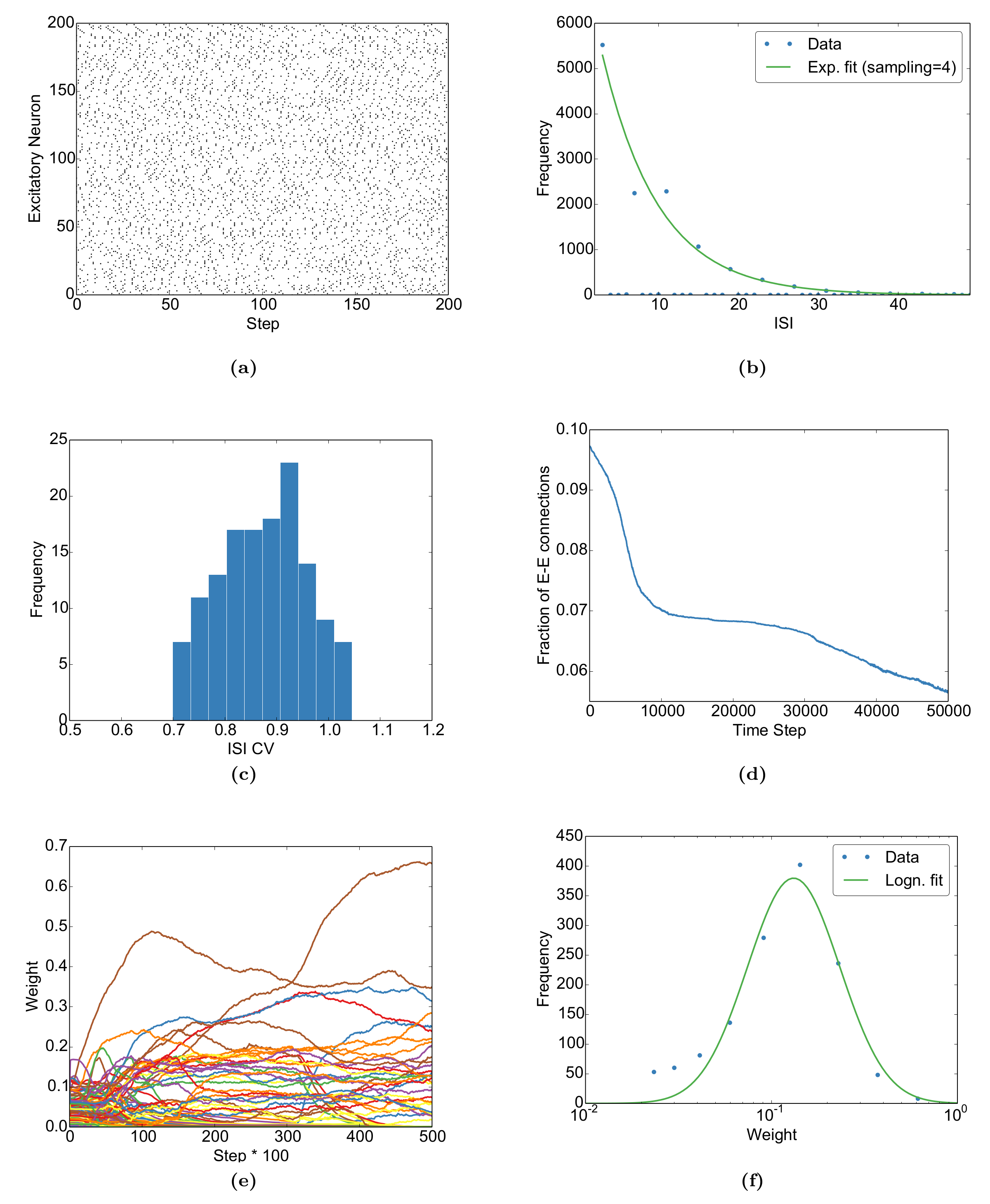

Supplement: S1 Fig — (a) A sample of spikes after plasticity. (b) The inter-spike-interval (ISI) distribution of a randomly selected neuron during spontaneous activity is well-fitted by an exponential after accounting for the periodic structure of the task. (c) The distribution of coefficients of variation (CVs) of the ISIs is on average slightly smaller than for the random letters (cp. Fig 1). (d) The fraction of excitatory-to-excitatory connections initially converges to a stable fraction, then decreases again. The network behaviour does not change despite the transient decrease. (e) Individual weights fluctuate despite the global periods of convergence. (f) After self-organization, the binned distribution of excitatory-to-excitatory synaptic weights (dots) is well fit by a lognormal distribution (solid line). (TIF) [file pcbi.1004640.s001.tif]

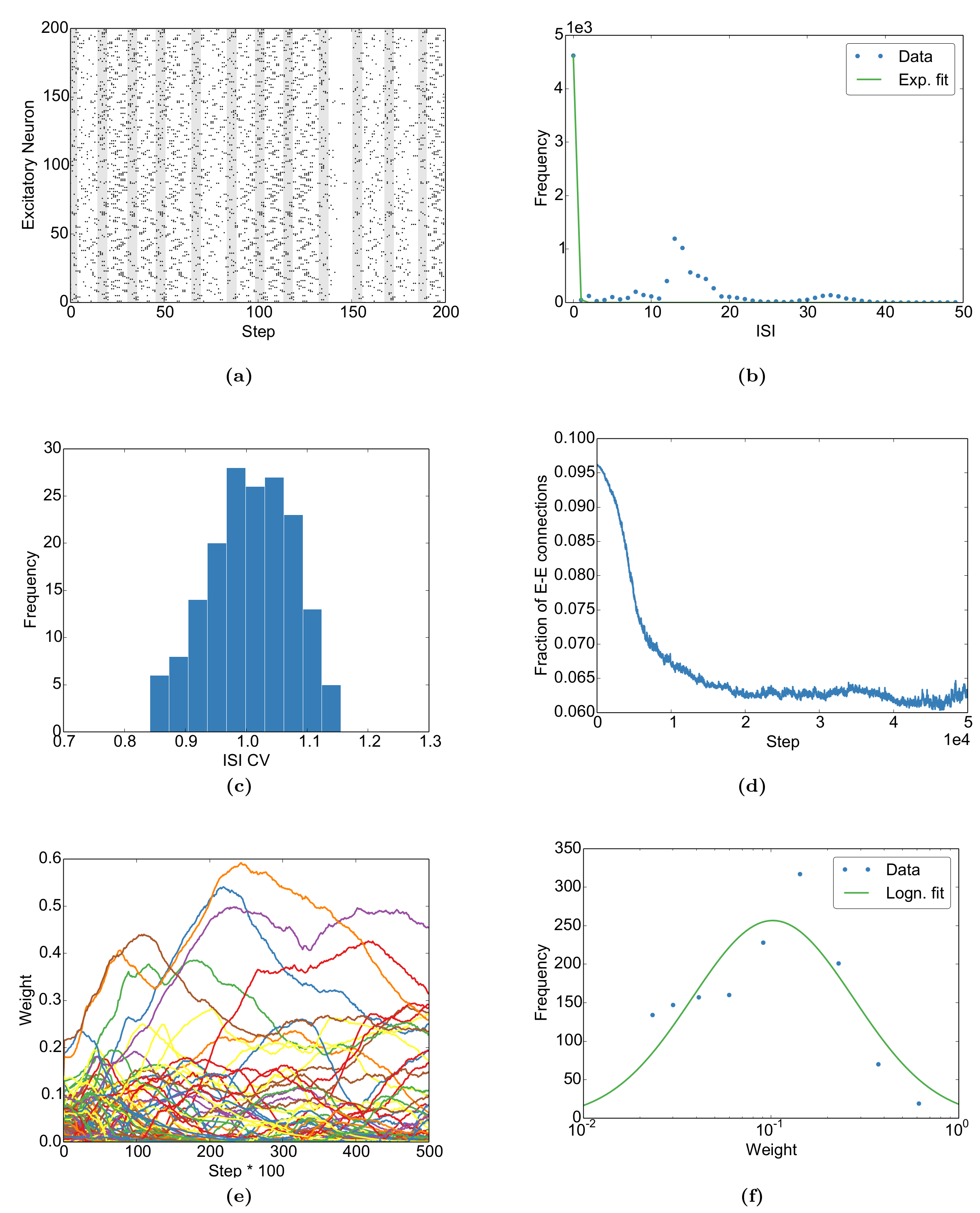

Supplement: S2 Fig — (a) A sample of spikes during the test phase. Stimuli were presented in the shaded areas. (b) The inter-spike-interval (ISI) distribution of a randomly selected neuron during spontaneous activity reflects the trial-like structure during learning with pauses between activity bursts. (c) The distribution of coefficients of variation (CVs) of the ISIs clusters around one. (d) The fraction of excitatory-to-excitatory connections converges to a stable fraction. (e) Individual weights fluctuate despite the global convergence. (f) After self-organization, the binned distribution of excitatory-to-excitatory synaptic weights (dots) deviates from a lognormal distribution (solid line). (TIF) [file pcbi.1004640.s002.tif]

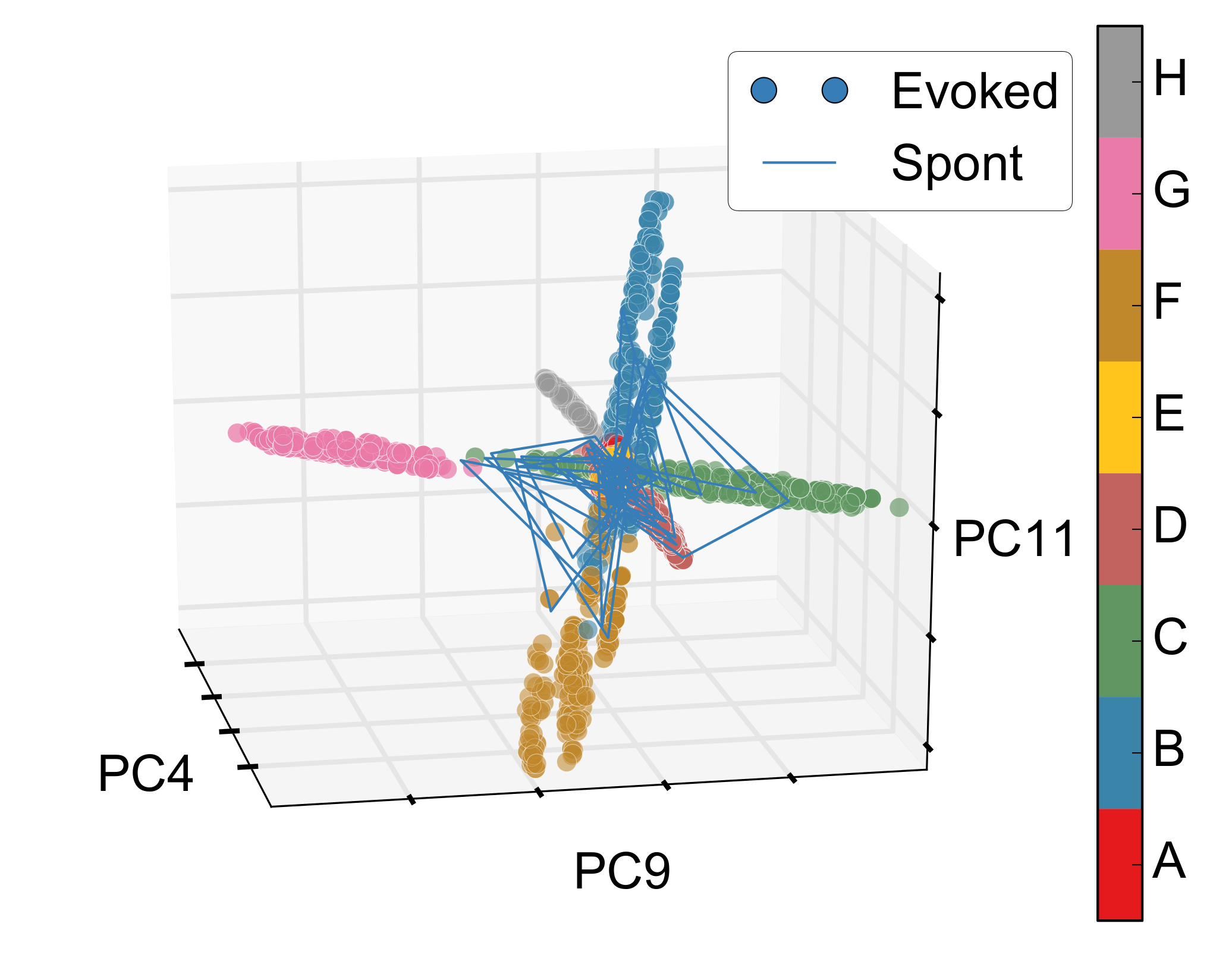

Supplement: S3 Fig — In the simulation of Fig 3a, the network was stimulated with the words “ABCD” (67%) and “EFGH” (33%) and evoked (dots) and spontaneous (lines) activity were projected into the principal component space of the evoked activity. Here, we display the 4th, 9th, and 11th principal component (PC) from that simulation. In each dimension, one letter of the two sequences clearly separates. The less variability is explained by the PC (i.e. the higher the PC number), the earlier the letter occurs in the sequence. As argued in the text, this effect might be due to small differences at the beginning accumulating through the recurrent structure to larger differences towards the end of the word. As expected from the other results in Fig 3, the spontaneous activity follows the structure of the sequences. For example, it transitions from blue to green (“B” to “C”) or from gold to pink (“F” to “G”) but not from blue to pink or form gold to green. This causes the opposing triangles of spontaneous activity in the plot. (TIF) [file pcbi.1004640.s003.tif]

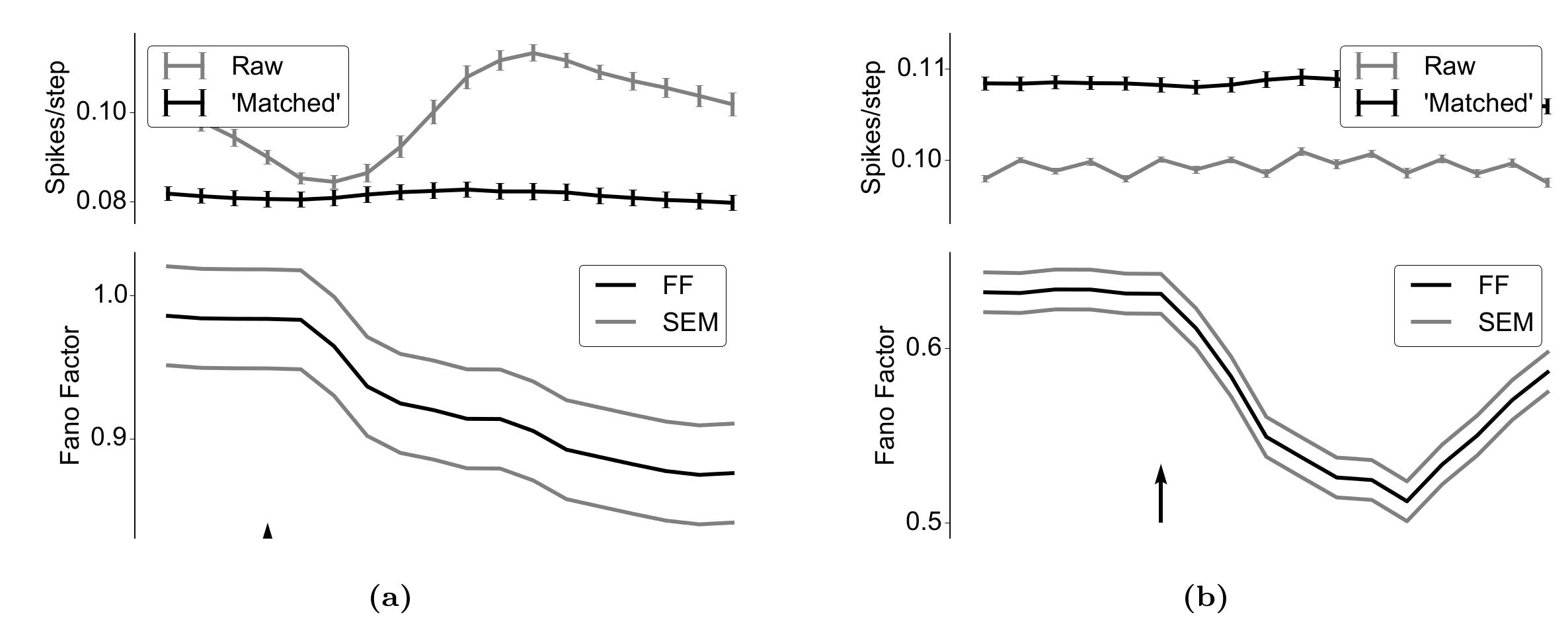

Supplement: S4 Fig — To control for effects of the mean, we computed the Fano factors with the mean-matching method proposed by [10]. This was done for both (a) the inference task and (b) the sequence learning task. As in the original paper, we averaged over all conditions. Therefore, we cannot distinguish between stimuli as we did in Fig 5. Both conditions used the same prior as in Figs 3 and 7. Please note that the sequence task does not have a trial structure but the network is continuously stimulated. Therefore, there is on average no rate change in raw activity at the onset of each stimulus. Error envelopes are SEM over 20 independent realizations. (TIF) [file pcbi.1004640.s004.tif]

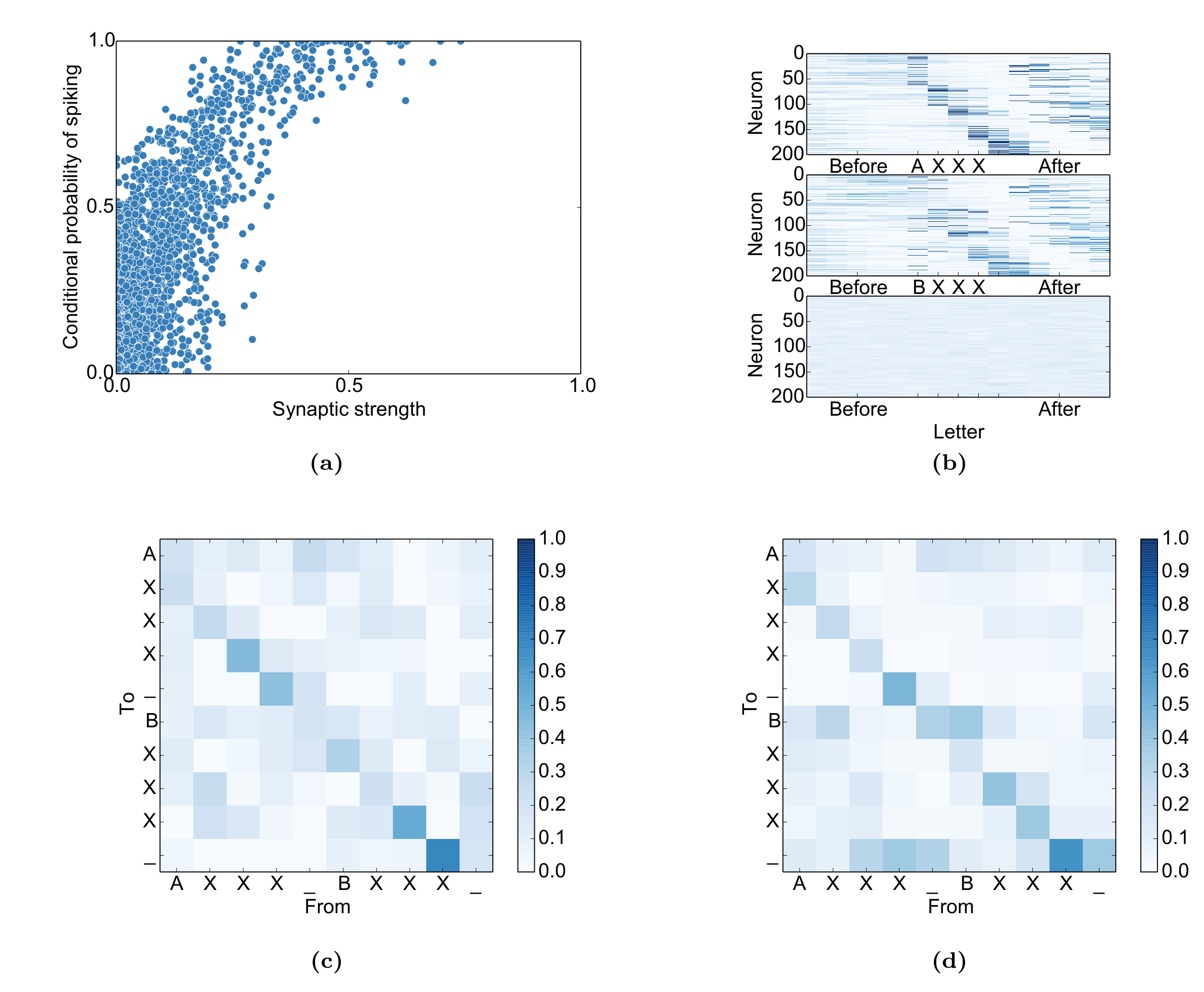

Supplement: S5 Fig — (a) The conditional probability of spiking p(x i(t + 1) = 1|x j(t) = 1) is directly proportional to its synaptic weight WijEE. (b) The firing probabilities of each neuron relative to stimulus onset. The network develops sequential activity patterns during the presentation of both sequences (top and middle). Neurons were sorted according to their maximal firing probability relative to the sequence “AXXX_ _ _ …”. (c) The prediction of transition probabilities during spontaneous activity from the singular value decomposition of W EE. (d) The actual transition probabilities during spontaneous activity. Please note the prominent “_” to “_” transitions reflecting the blank periods during training. (TIF) [file pcbi.1004640.s005.tif]

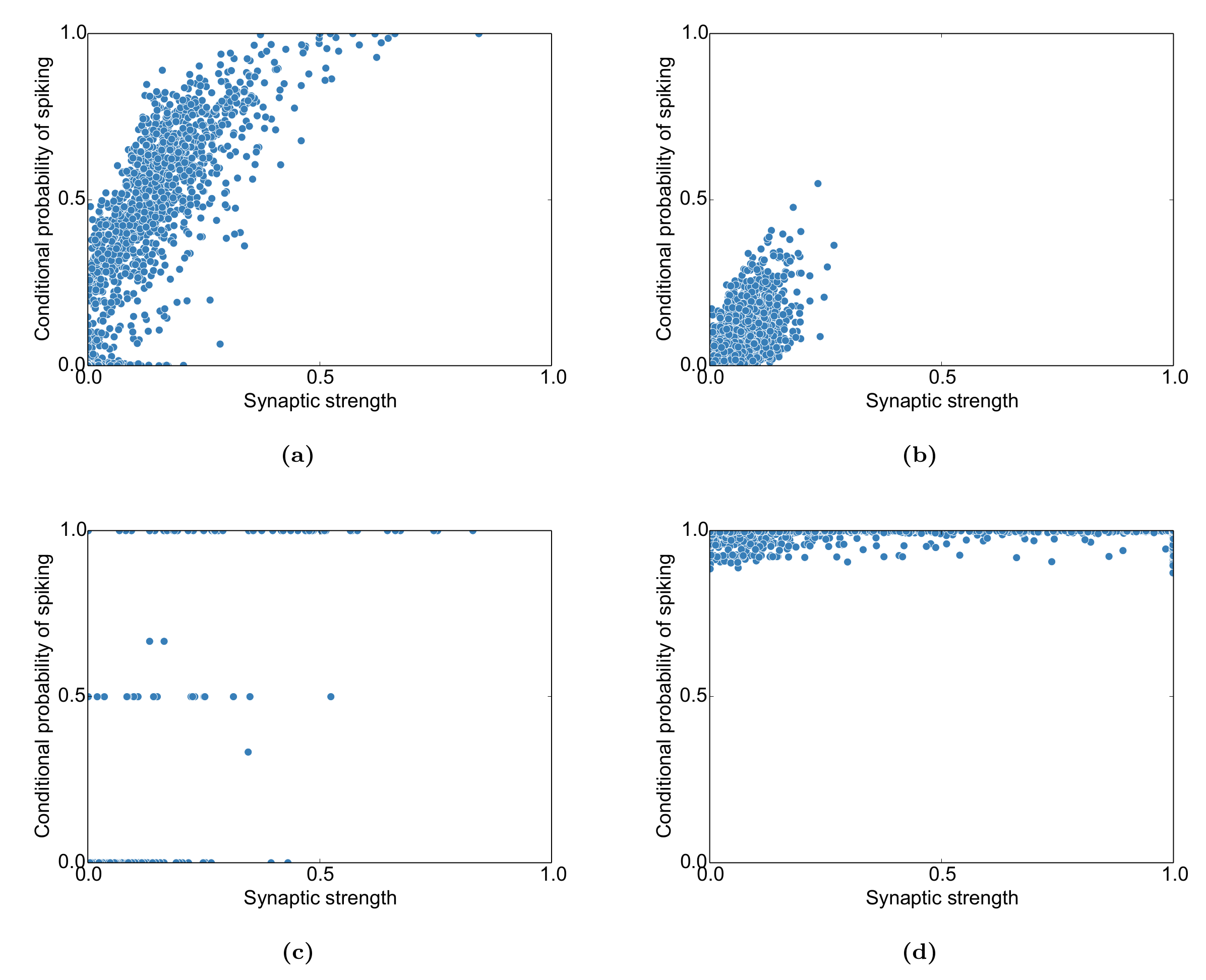

Supplement: S6 Fig — (a) Conditional firing probabilities from Fig 8a. (b) The original simulation without STDP demonstrates that STDP is not necessary for the linear relation between synapse strength and firing probability. Also, STDP leads to stronger weights. (c) The original simulation without IP shows that IP is essential for maintaining correct firing probabilities. (d) The same holds true for a simulation without synaptic normalization. Both (c) and (d) also show incorrectly learnt weight matrices and pathological network dynamics (see [37] for effects of excluding IP or SN on network dynamics). (TIF) [file pcbi.1004640.s006.tif]

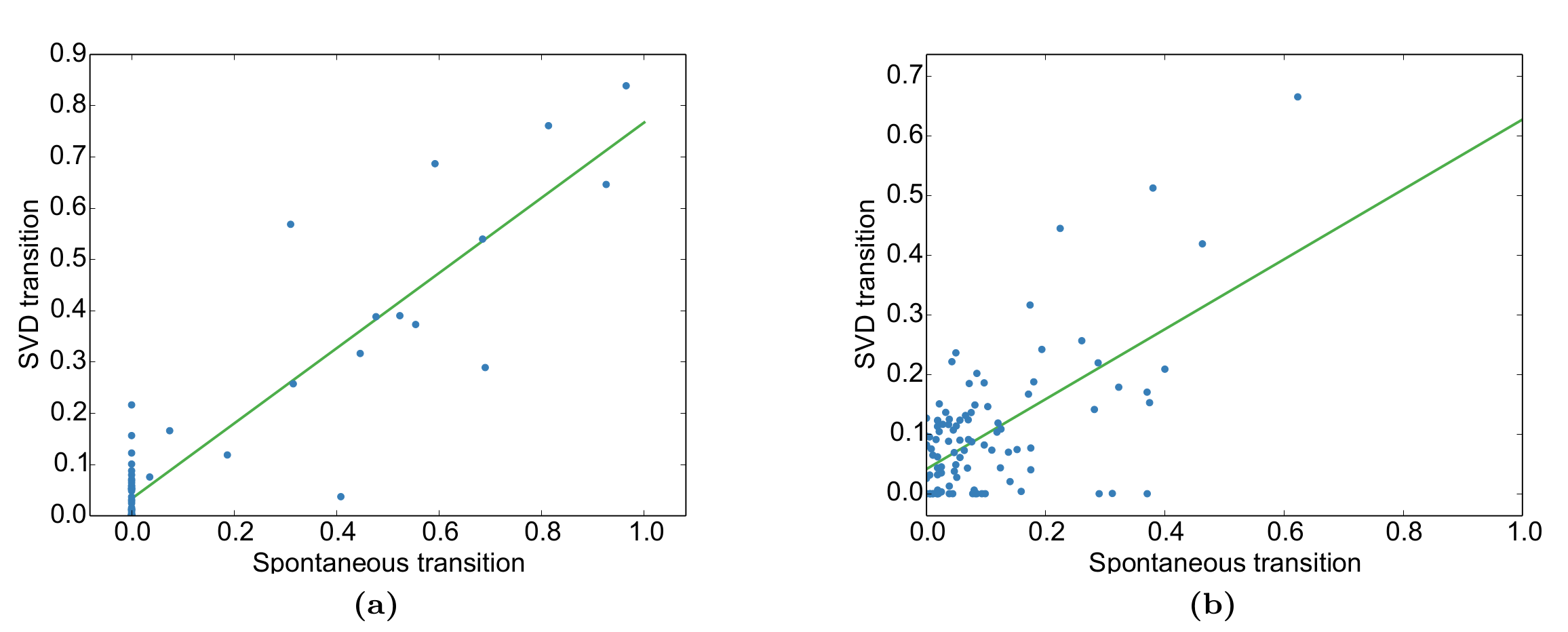

Supplement: S7 Fig — (a) The transition probabilities between letters during spontaneous activity and its estimates from singular value decomposition are plotted for the sequence learning task. The green line is the fitted linear regression. The individual transition probabilities are shown in Fig 8c and 8d. (b) The same plot for the data from the inference task (S5c and S5d Fig) shows a worse match. This is due to more spontaneous activity during training and the thereby less constrained excitatory weight matrix. This results both in smaller transition probabilities during spontaneous activity and a more ambiguous SVD analysis of the weight matrix. (TIF) [file pcbi.1004640.s007.tif]
